# Structures of EHD2 filaments on curved membranes provide a model for caveolar neck stabilization

**DOI:** 10.1038/s41467-026-76288-8

**Published:** 2026-07-31

**Authors:** Elena Vázquez-Sarandeses, Vasilii Mikirtumov, Jeffrey K. Noel, Mikhail Kudryashev, Oliver Daumke

**Affiliations:** 1 Structural Biology, Max Delbrück Center for Molecular Medicine, Berlin, Germany; 2 Institute of Chemistry and Biochemistry, Freie Universität Berlin, Berlin, Germany; 3 Institute of Medical Physics and Biophysics, Charité-Universitätsmedizin Berlin, Berlin, Germany

**Keywords:** Cryoelectron tomography, GTP-binding protein regulators, Lipid signalling, Membrane structure and assembly

## Abstract

Caveolae are flask-shaped invaginations of the plasma membrane serving critical functions in mechano-protection and signal transduction. Caveolar dynamics, such as caveolar movement within the plasma membrane or endocytosis, relies on precise shaping of the highly curved caveolar necks. The dynamin-like EHD2 ATPase is proposed to oligomerize around the caveolar neck, but its detailed molecular action is poorly understood. Here, we employ cryo-electron tomography to elucidate structures of ring-like EHD2 filaments on tubulated liposomes. EHD2 forms highly curved membrane scaffolds which stabilize a tubular membrane geometry with undulations along the tube’s axis, resembling caveolar neck architecture. An amino-terminal sequence facilitates this geometry by acting as a spacer between adjacent filaments. Moreover, in endothelial cells lacking EHD2, caveolar necks become narrower and more elongated. Our structural work provides the molecular framework for understanding EHD2 scaffold formation and its cellular function in caveolar dynamics.

## Abstract (web summary)

Caveolae rely on specialized protein scaffolds to maintain their characteristic membrane shape and function. Here, authors show dynamin-like EHD2 assembles into ring-like filaments that stabilize caveolar necks and identify an amino-terminal spacer that controls scaffold architecture.

## Introduction

The family of Eps15-homology domain-containing proteins (EHDs) are dynamin-related ATPases exclusively found in eukaryotes^1^. The four mammalian EHD proteins have been associated with diverse cellular processes that require membrane remodeling and/or preservation of specific membrane shape (reviewed in ref. ^2^): EHD1 mediates endocytic recycling by regulating vesicle fission and membrane trafficking^3–6^. Together with Rab11FIP5, rabenosyn-5, VPS45, and VIPAS39, EHD1 forms the FERARI complex, which coordinates membrane fusion and fission in the Rab11-dependent recycling pathway^7^. EHD1, in cooperation with EHD3, also participates in ciliogenesis^8^. Furthermore, EHD1 cooperates with the Bin-Amphiphysin-Rvs167 (BAR)-domain containing BIN1 protein during endocytic recycling^5^ and during the formation of the T-tubule network in muscle^9,10^. Deletion of EHD3 in mice leads to enlarged hearts and abnormal cardiac function^11^. In neurons, EHD4 was shown to localize to macropinosomes^12^, mediating TrkA receptor uptake via macropinocytosis^13^. In non-neuronal cells, EHD4 regulates EHD1-mediated endosomal recruitment and vascular endothelial (VE)-cadherin membrane trafficking^14,15^.

EHD2 accumulates at injury sites in human myotubes and assists in the membrane repair process^16,17^. The best characterized function of EHD2, however, is linked to its localization at caveolae^18–25^. Caveolae are small plasma membrane invaginations involved in mechano-protection and control of lipid homeostasis^26,27^. EHD2 was proposed to stabilize caveolae by oligomerizing around their necks^18,19,25^. Mutations in EHD2 interfering with membrane binding, oligomerization, nucleotide binding or hydrolysis^28^ yield abnormal caveolar morphologies^18,21^. In a mouse model deficient for EHD2, various cell types display detached caveolae and increased caveolar mobility^22,24^, closely phenocopying the effect of EHD2 knockdown in cell culture^18^. Accordingly, it was suggested that EHD2 restricts lateral diffusion and detachment of caveolae from the plasma membrane. In the EHD2 knockout mice, adipocytes were enlarged and contained more and expanded lipid droplets, concomitant with increased visceral fat deposits at several organs^24^. Furthermore, in mesentery arteries, EHD2-dependent caveolae stabilization is required for mesentery relaxation via the endothelial nitric oxide synthase pathway^29^. Collectively, EHD2 appears to be a critical component for maintaining caveolae-associated signaling and uptake functions.

Biochemical and structural analyses provided insights into the mechanism of EHDs in membrane remodeling. The GTPase (G-) domain of EHDs surprisingly binds to adenine rather than guanine nucleotides^28,30^. When incubated with liposomes, EHDs form ATP-dependent oligomeric assemblies at the membrane surface, leading to liposome tubulation^4,5,20,28,31,32^. In the membrane-bound, oligomeric state, the slow ATPase activity of EHD proteins is moderately stimulated^4,28,31^. EHD1, but not EHD2, was shown to cleave preformed membrane tubules in an ATPase-dependent manner^4^.

**Fig. 1:**
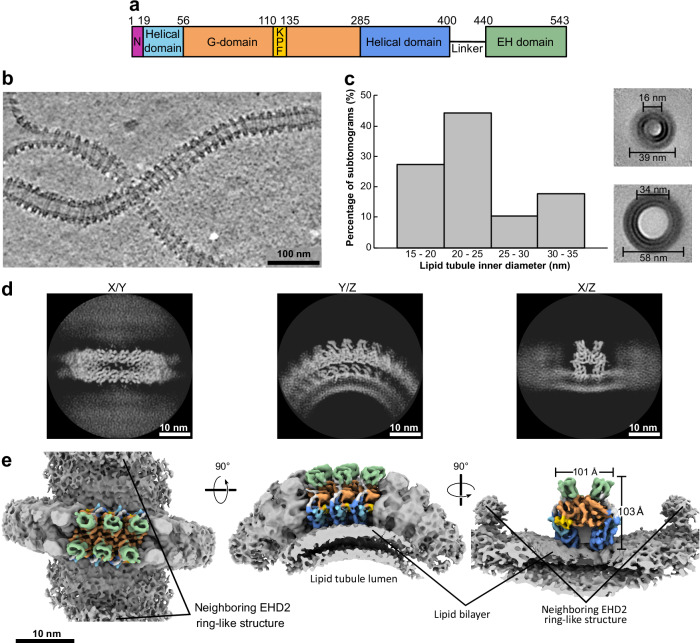
Structure determination of membrane-bound EHD2. **a** Domain architecture of EHD2. Residue numbers refer to the mouse sequence. **b** Central slice of a representative cryo electron tomogram of lipid tubules decorated with EHD2 ring-like oligomers. **c** Distribution of particles according to the lipid tubules’ inner diameter. The lumen of the tubules, as measured in cross-sections of full 2D projections of subtomogram averages, is within a range of 16–34 nm. **d** Projections of the resulting subtomogram average map of membrane-bound full-length EHD2. The view axis is indicated on top of each panel. **e** Surface representation of the subtomogram average map. The view axis is the same as the panels above in (**d**). The asymmetric unit, resolved at an average resolution of 6.7 Å, is colored according to (**a**).

Crystallographic structural analyses indicated that the G-domain mediates stable dimerization via a conserved interface in the dynamin superfamily (Supplementary Fig. 1). The adjacent helical domain is composed of sequences preceding and following the G-domain. It contains the primary membrane-binding site at its tip^28^. The helical domain is connected via a long linker to the C-terminal EH domain (Fig. 1a), which mediates binding to linear peptide motifs containing an NPF (Asn-Pro-Phe) motif^28,33^. In the crystal structure of the dimer, each EH domain is positioned on top of the opposing G-domain and binds to the Gly-Pro-Phe (GPF) motif in the linker region between the helical and EH domains. The two helical domains protrude in parallel, representing the “closed” conformation of EHDs (Supplementary Fig. 1a)^28^.

The crystal structure of an N-terminally truncated variant of EHD4 (EHD4^ΔN^) features a large-scale 50° rotation of the helical domain relative to the G-domain compared to the EHD2 crystal structure. This arrangement was termed the “open” conformation (Supplementary Fig. 1b)^31^. While spectroscopic studies suggested that EHD2 recruitment to membranes occurs in the open state^21^, a cryo-electron tomography (cryo-ET) derived subtomogram averaging (STA) structure indicated that membrane-bound EHD4^ΔN^ filaments feature a close conformation of the dimeric building block^32^. Accordingly, it was proposed that EHDs exist as open dimers in solution and undergo a conformational rearrangement to the closed state when recruited to membranes and assembled into membrane-bound oligomeric filaments.

In the EHD4^ΔN^ filaments, oligomerization is mediated via three distinct interfaces: Interface-1 represents the EHD-specific dimerization interface also found in the open and closed dimeric crystal structures. Interface-2 involves a KPF (for Lys-Pro-Phe)-containing loop in the G-domain that assembles with the helical domain of the adjacent dimer^31,32^. A conserved G-domain interface across the nucleotide-binding site (the G-interface) constitutes interface-3. Oligomeric EHD4^ΔN^ assembled into filaments with a low spontaneous curvature of ~1/70 nm^−1^ (ref. ^32^). In contrast, EHD2 oligomerizes at membranes into ring-like structures of much higher curvature^28^. The molecular basis of the different assembly types in EHD4 and EHD2 is not known.

In the crystal structure of EHD2, a conserved N-terminal sequence stretch binds into a hydrophobic pocket at the periphery of the G-domain (Supplementary Fig. 1)^20^. Electron paramagnetic resonance experiments indicated that the N-terminal stretch moves from the pocket in the G-domain into the membrane, representing a secondary membrane binding site^20^. In turn, the KPF loop substitutes for the N-terminus in the hydrophobic G-domain pocket to generate interface-2^31,32^. The N-terminal switch was suggested to regulate EHD2 oligomerization at the caveolar neck^20,21^. Also in EHD1, the deletion of the N-terminal residues caused defects in the stability of the membrane scaffold and affected its endocytic recycling activity^4^. However, the structural role of the N-terminal sequence and its regulatory function have remained unclear since the N-terminus was not fully resolved in the EHD2 crystal structures^20,28^ and was absent in the EHD4 construct used for crystal and STA-cryo-ET structure determination^31,32^.

In the current study, we used cryo-ET and STA to determine the structure of membrane-bound full-length EHD2 and an N-terminal truncated variant (EHD^ΔN^) reconstituted on highly curved lipid tubes. Compared to the previously described EHD4^ΔN^ filaments, we observe distinct arrangements of EHD2 and EHD2^ΔN^ filaments on lipid tubes. The resolution of our STA structures allowed the fitting of available crystal structures, including key oligomerization elements, providing insights into the assembly and membrane remodeling mechanism. The higher curvature of the EHD2 versus the EHD4^ΔN^ scaffold is achieved by a different assembly angle between the dimeric building blocks. By comparing the EHD2 and EHD2^ΔN^ filaments, we uncover a crucial role of the N-terminal sequence as a spacer, allowing the formation of a ring-like EHD2 scaffold stabilizing high membrane curvature at the caveolar neck.

## Results

### Structure determination of membrane-bound EHD2

We previously reported that mouse EHD2 binds to liposomes composed of a bovine brain lipid extract (e.g., Folch liposomes) and deforms them in an ATP-dependent fashion into tubules by forming oligomeric ring-like structures around them^28^. To structurally characterize the EHD2 assembly mode, we purified full-length mouse EHD2 (Fig. 1a) and reconstituted it on Folch liposomes. Using cryo-ET, we observed that EHD2 coated and occasionally tubulated liposomes in the absence of ATP, in line with our previous report^20^. However, EHD2 failed to arrange into regular filaments in the absence of nucleotide (Supplementary Fig. 2a). In the presence of ATP, membrane tubulation was massively increased, and ring-like EHD2 assemblies were found, confirming the ATP requirement for oligomerization (Fig. 1b and Supplementary Fig. 2b–e). In samples vitrified 2 h after their reconstitution with ATP (at this timepoint, about 90% ATP is hydrolyzed, ref. ^28^), oligomer coating was disrupted on most tubules, which often displayed an irregular, thin appearance (Supplementary Fig. 3a). This is in line with our previous suggestions that ATP hydrolysis in EHD4^ΔN^ leads to oligomer disassembly^32^ and highlights the scaffolding role of EHD2 in stabilizing membrane curvature.

We then collected 95 tilt-series of the ATP- and membrane-bound EHD2 filaments using a hybrid dose scheme with a high dose image at 0° tilt^34^. In addition to the ring-like structures on highly curved lipid tubules (Fig. 1b and Supplementary Fig. 2b, c), EHD2 also formed short oligomeric filaments on the surface of non-tubulated liposomes featuring low membrane curvature (Supplementary Fig. 2b, d). At transition areas, where lipid tubules emerged from the liposomes, short EHD2 filaments approached each other (Supplementary Fig. 2b, e), possibly in the process of oligomerizing into ring-like assemblies, triggering membrane curvature.

To structurally characterize the membrane-bound EHD2 rings, we employed STA by defining subtomograms along the axis of the lipid tubules (Supplementary Fig. 4). Alignment and classification of the tubules revealed that their luminal diameters ranged from 16 to 34 nm (Fig. 1c and Supplementary Table 1). These diameters are significantly smaller than those of the membrane tubes formed by EHD4^ΔN^, which were in the range of 30–100 nm (ref. ^32^). Several rounds of subtomogram classification and “subboxing”^35^ resulted in 75,439 lipid tube segments from an initial set of 14,491 subtomograms (Supplementary Fig. 4a). The final structure was refined by focusing on a segment within one ring, yielding an average resolution of 6.7 Å, locally ranging from 6.2 to 9.4 Å (Fig. 1d, e, and Supplementary Fig. 4b–f). At this resolution, secondary structure elements could be clearly discerned in the map, while amino acid side chains were not resolved. The asymmetric unit of the STA structure includes six monomers of EHD2 arranged into two dimers and two monomers corresponding to adjacent dimers within the filament (Fig. 1e, Supplementary Fig. 4, and Supplementary Table 2). G-, helical and EH domains could be unambiguously identified in the map: while the G-domains localize to the core of the filament, the helical domains extended towards the membrane and the EH domains are located on top of the G-domains (Fig. 1e).

### Architecture of EHD2 filaments

**Fig. 2:**
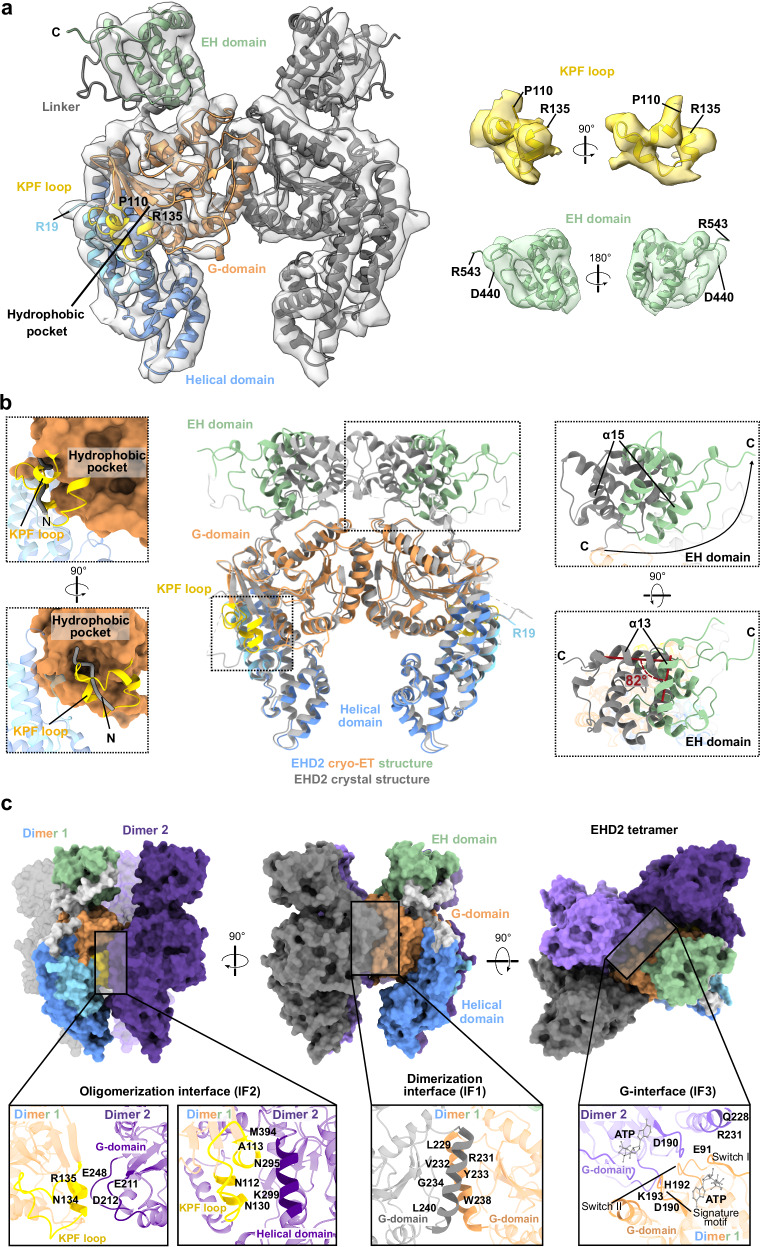
Structure of the EHD2 filaments. **a** Model of dimeric EHD2 fitted into the cryo-ET density. One monomer is colored according to the domains, and the other monomer is shown in gray. On the right, the fitting of the KPF loop and the EH domain in the density is highlighted. N- and C-terminal residues of these domains are shown to indicate their orientation. C-C-terminus. **b** Superposition of the cryo-ET EHD2 structure (colored, only one dimer is shown) and the dimeric EHD2 crystal structure (gray, PDB: 4CID). Structural differences are highlighted and magnified in the dotted rectangles. The EH domain undergoes a large-scale rotation of ~80°, which repositions the C-terminal tail to the outside of the oligomeric filament, thereby increasing the space between the EH domains in the EHD2 dimer. The KPF loop occupies the hydrophobic pocket of the G-domain (surface representation in the dotted rectangle), in which the N-terminus (gray) is buried in the EHD2 crystal structure. **c** Three interfaces drive oligomeric assembly and are highlighted in the central tetramer of the asymmetric unit. One dimer is formed by the monomer colored according to the domains and the monomer shown in gray. The other dimer is shown in two shades of purple. Front, side, and top views correspond to the left, middle and right panels. Highlighted panels are magnified. The dimerization interface involves the G-domains (interface-1, IF1). The oligomerization interface (interface-2, IF2) is established between the KPF loop of one monomer and the helical and G-domains of the neighboring monomer from the adjacent dimer. The canonical G-interface (interface-3, IF3) is formed between the nucleotide binding pockets of opposing monomers from adjacent dimers. The approximate positions of highly conserved residues in the interfaces (see also ref. ^28^) are indicated to highlight their potential involvement in the assembly.

A flexible fitting approach with the high-resolution EHD2 crystal structure was used to generate an atomic model of the EHD2 oligomer (Supplementary Fig. 5a, and see “Methods” for details). The G-domain covering residues 56–284 and the helical domains spanning residues 1–55 and 286–399 could be confidently modeled into the density (Fig. 2a). Clear density was also evident for the KPF loop (residues 110–135), which occupied a hydrophobic pocket in the G-domain (Fig. 2a). In the auto-inhibited EHD2 crystal structure, the N-terminus is buried in this pocket (Fig. 2b and Supplementary Fig. 1a). Similar to membrane-bound EHD4^ΔN^, oligomerized EHD2 was in the closed conformation (Fig. 2a and Supplementary Fig. 1a).

In contrast to the G-domain and helical domain, the EH domains were more flexible and, consequently, the corresponding densities had a lower resolution of 7–8 Å (Supplementary Fig. 6). To obtain a reliable fitting, we calculated correlation scores of an EH domain model in 70 rotations equally distributed around a unit sphere with the excised EH domain map (Supplementary Fig. 6a). In the model with the highest correlation score, the C-terminal tail pointed towards the outside of the filament (Fig. 2a, b, and Supplementary Fig. 6a, b). Contacts of EH domain helices α14 and α15 with the underlying G-domain stabilize the EH domain conformation in the oligomeric assembly (Supplementary Fig. 6c). Comparison of the EHD2 crystal structure and the STA-cryo-ET-based model revealed that the EH domains undergo a large-scale ~80° rotation upon oligomerization, resulting in a shift towards the periphery of the filament (Fig. 2b). No density was apparent for the long, disordered linker between the EH domain and the helical domain, leading to an ambiguity in the connection of the domains. We assigned the EH domain to the helical domain situated directly below, since the connection of the domain termini was the shortest in this way.

Three interfaces were previously defined in the EHD4^ΔN^ oligomer^32^, which we also found in the EHD2 oligomer (Fig. 2c). The resolution of the cryo-EM map did not allow an accurate assignment of side chains, but with the EHD2 crystal structure as a basis, we included them in the model to highlight possible molecular interactions (Fig. 2c and Supplementary Fig. 4).

Interface-1 mediates EHD2 dimerization via the EHD-specific interface and mainly involves helices α6 from the G-domains of opposing monomers, which assemble in a symmetric fashion (Fig. 2c). Interface-2 and 3 drive the oligomerization of EHD2 dimers. Oligomerization interface-2 involves the KPF loop of one protomer and helices α8 and α12 from the helical domain of the adjacent dimer. Additional contacts not described for the EHD4^ΔN^ oligomer are formed between the KPF loop of one protomer and two short loops from the opposing G-domain (Fig. 2c). The canonical G-interface, designated interface-3, is built between residues surrounding the nucleotide binding pockets of opposing G-domains from adjacent dimers (Fig. 2c). The interface involves highly-conserved surface-exposed loops, such as switch I and II and the EHD-specific signature motifs. In analogy to other dynamin-related proteins^36^, this interface is likely responsible for the ATP-dependency of the assembly.

### The N-terminus acts as a membrane-embedded spacer between filaments

**Fig. 3:**
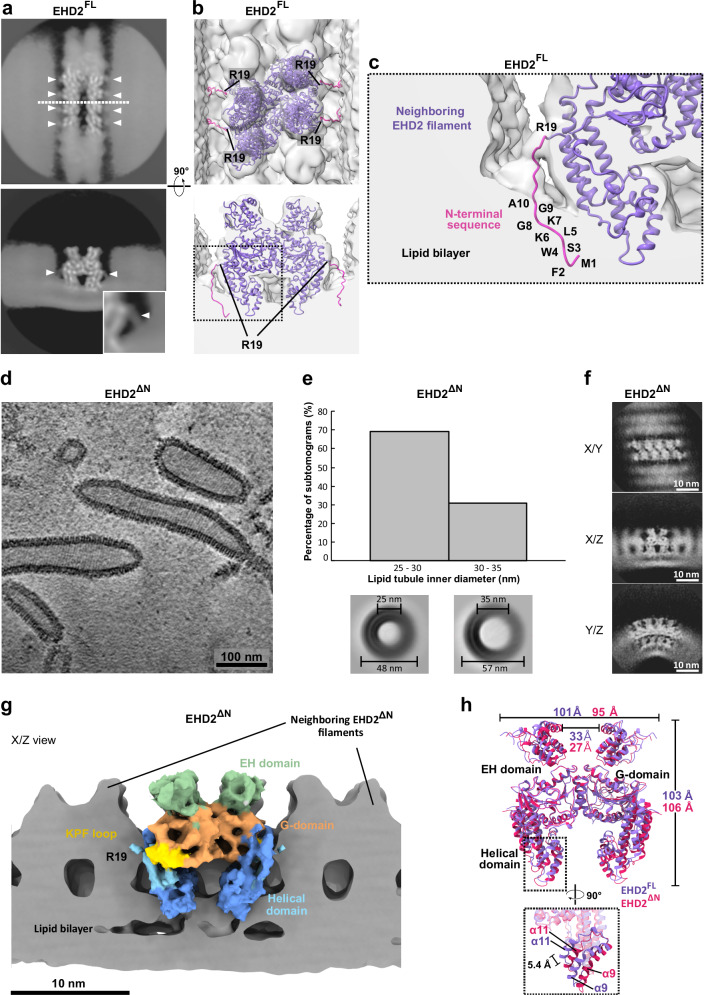
The N-terminus acts as a spacer between filaments. **a** Unsharpened subtomogram averaging map reveals a pattern of low-resolution densities at the periphery of the EHD2 filament (white arrowheads and magnified inlet). Top: Z view, bottom: Y view clipped at the white dashed line. C-C-terminus. **b** 3D surface representation of the panels shown in (**a**) with the central EHD2 tetramer fitted in the density. The low-resolution density emerges from the first modeled residue (Arg19) and reaches towards the lipid bilayer. The N-terminal peptide (magenta) is sufficiently long to cover the distance to the lipid bilayer and insert. **c** Magnified view of the inlet highlighted in (**b**). Despite weak density, the N-terminal loop was included in the model to show that it can reach the membrane. Hydrophobic and positively charged residues may insert into the outer leaflet of the lipid bilayer in proximity of the neighboring EHD2 filament. **d** The absence of the N-terminus results in tightly packed oligomeric filaments around the lipid tubules. A central slice of a representative tomogram is shown. **e** Distribution of particles according to lipid tubule inner diameter, measured in cross-sections of full 2D projections of subtomogram averages. Lipid tubules generated by EHD2^ΔN^ are more homogenous in their lumen diameters compared to full-length EHD2. **f** Projections of the resulting subtomogram average map of membrane-bound EHD2^ΔN^, obtained at an average resolution of 10.1 Å. Neighboring filaments in close proximity are observed in the X/Y and X/Z views. **g** Surface representation of the subtomogram average map. View axis (X/Z) as in the middle panel of (**f**). The central tetramer is colored according to the domains. **h** Superposition of full-length EHD2 (purple) and EHD2^ΔN^ (magenta) cryo-ET models. N-terminal deletion results in a slightly higher structure, and the EH domains approach each other. Helices α9 and α11 from the helical domain are displaced by 5.4 Å (magnified). The disordered linker between the helical and the EH domains is hidden for better visualization.

When examining an unsharpened map, a distinctive density pattern was found next to the first ordered residue (around Arg19) and reached towards the lipid bilayer while approaching the neighboring EHD2 filaments (Fig. 3a). In agreement with previous electron paramagnetic spin resonance (EPR) data indicating membrane interaction of the N-terminus^20^, we assigned this density to the N-terminal sequence and included it in our model. Notably, the N-terminal peptide is sufficiently long to cover the length of the density and to insert with its N-terminal conserved hydrophobic and positively charged residues into the lipid bilayer (Fig. 3a–c).

To further characterize the role of the N-terminus for the assembly, we reconstituted an N-terminally truncated EHD2 construct lacking the first 18 amino acids (residues 19–543, EHD2^ΔN^) on liposomes. We noticed that EHD2^ΔN^ filaments formed a more tightly packed coat on the surface of lipid tubes compared to full-length EHD2 (Fig. 3d, compare to Fig. 1b). Accordingly, EHD2^ΔN^-coated lipid tubes were more homogenous in size, featuring tubular membrane diameters of 25–35 nm (Fig. 3e).

Employing the same experimental setup as for full-length EHD2, we collected 110 tilt series for EHD2^ΔN^-covered lipid tubes (Supplementary Table 2). We then determined the structure of EHD2^ΔN^ at an average resolution of 10.1 Å by STA using 17,204 particles subboxed from an initial set of 30,449 subtomograms (Supplementary Fig. 7). The local resolution ranged from 6.3 to 16.6 Å, with the EH domain being the most flexible and therefore less well resolved domain (Supplementary Fig. 7). The asymmetric unit includes eight monomers of EHD2^ΔN^ which form four dimers (Fig. 3f, Supplementary Table 2, and Supplementary Fig. 7). Similar to the EHD2 filaments, the EHD2^ΔN^ model was generated by using a molecular dynamics simulations-based map fitting approach with geometric restraints from the dimeric EHD2 crystal structure and the previously determined oligomeric full-length EHD2 as a starting model (Supplementary Fig. 5b, and see “Methods” for details).

As expected, the low-resolution density corresponding to the N-terminal residues in the EHD2 filaments was absent in the EHD2^ΔN^ map (Fig. 3g). The conformations of EHD2 and EHD^ΔN^ appeared overall similar in the filaments. Minor structural rearrangements of α9 and α11 in the helical domains were observed, and the EH domains moved 6 Å closer to each other in EHD^ΔN^ compared to EHD2 (Fig. 3h). Also, the assembly mode of the EHD2^ΔN^ filaments via the three oligomerization interfaces was not perturbed.

**Fig. 4:**
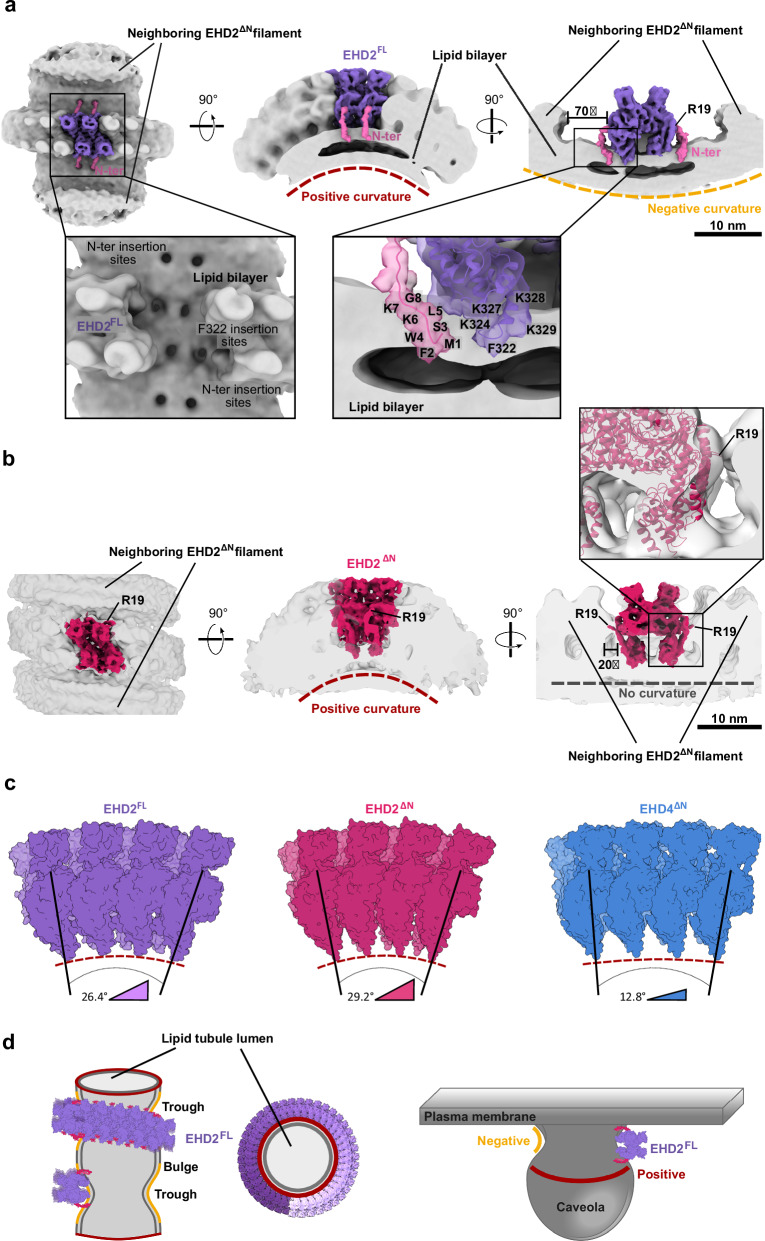
EHD2 filaments stabilize a membrane geometry reminiscent of the caveolar neck. **a** 3D surface representation of the subtomogram average map, including the lipid bilayer. The central tetramer of the asymmetric unit is highlighted in purple. The modeled N-terminus with its surrounding low-resolution density map is displayed in pink. Left: top view of an EHD2 ring-like structure. Middle: side view showing positive curvature of the membrane tubule stabilized by the EHD2 filament. Right: front view showing negative membrane curvature in undulations along the tubule’s axis. Magnified inlets show how EHD2 inserts the tip of the helical domain and the first residues of the N-terminus in the outer leaflet of the lipid bilayer. Left: The central tetramer is hidden to show how the helical domain and the N-terminus penetrate into the membrane. Right: residues potentially involved in membrane binding are indicated in one monomer. **b** 3D surface representation of the EHD2^ΔN^ subtomogram average map, including the lipid bilayer. The central tetramer of the asymmetric unit is highlighted in magenta. Left: top view showing oligomeric filaments in close vicinity. Middle: side view showing positive membrane curvature. Right: the deletion of the N-terminus renders the lipid bilayer flat. The magnified inlet shows the absence of extra densities emerging from Arg19, which points toward the neighboring filament. **c** EHD2 and EHD2^ΔN^ filaments (purple and magenta, respectively) are more curved than EHD4^ΔN^ filaments (blue, PDB: 7SOX). The dashed red lines indicate the positive curvature of the lipid bilayer. The side view of an octamer is shown for each protein. **d** Schematic representation of the in vitro membrane tubulation activity of EHD2 (left) and its relation to the suggested localization at the neck of caveolae (right), where positive and negative membrane curvature co-exist in a similar fashion.

Importantly, EHD2^ΔN^ filaments assembled into tightly packed filaments rather than ring-like structures (Fig. 3d, compare to Fig. 1b). Similar to the EHD4^ΔN^ filaments, peripheral helices α1a, α1b, and α2 in the G-domain contacted each other across adjacent filaments (Fig. 3f, g), whereas they were 70 Å apart in the EHD2 full-length assemblies (Fig. 4a, right). These observations indicate a function of the membrane-inserted N-terminus as a spacer between adjacent filaments required for the formation of distinct ring-like structures.

### EHD2 filaments stabilize a tubular membrane geometry with undulations

Clear density for the lipid bilayer allowed us to characterize the membrane-binding mode of EHD2 (Fig. 4). In line with previous EPR data^20^, a loop at the tip of the helical domain containing Phe322 inserts into the outer leaflet of the lipid bilayer which would be expected to induce membrane curvature by increasing the surface area of the outer membrane leaflet (Fig. 4a). In addition, positively charged residues, such as Lys324, and Lys327-329, which were previously shown to contribute to membrane binding, are in close contact to the bilayer (Fig. 4a) and likely mediate the phosphatidyl-inositol(4,5)bisphosphate specificity of EHD2 (refs. ^28,31^). Moreover, the proposed position of the N-terminal stretch close to the membrane (Figs. 3a–c, 4a) supports its role as a secondary membrane-binding site^20^ (Fig. 4a).

In the EHD2 full-length filaments, the membrane surface along the tubule’s axis showed undulations, with the EHD2 filament sitting in the troughs of the undulations (Fig. 4a, d). In this way, positive and negative membrane curvature is generated along the tubule’s axis (Fig. 4a, d). In contrast, no undulations were observed for EHD2^ΔN^ filaments, resulting in a flat lipid bilayer along the tube axis (Fig. 4b). Notably, the membrane geometry stabilized by EHD2 filaments resembles the membrane architecture of the caveolar neck, where also a combination of positive and negative membrane curvature is found (Fig. 4d).

The angle between two assembling EHD2 dimers was ~26° (Fig. 4c). The N-terminal deletion did not grossly alter the assembly angle between two adjacent dimers (~26° for full-length EHD2 versus ~29° for EHD2^ΔN^) resulting in a similar diameter of the underlying membrane tube (Fig. 4c). In stark contrast, the assembly angle was only ~13° in the EHD4^ΔN^ filaments^32^. The higher assembly angle leads to the observed G-domain-G-domain contacts involving the KPF-loop (Fig. 2c). It also underlies the smaller diameter of EHD2 rings, which, accordingly, stabilize higher membrane curvature compared to EHD4^ΔN^ filaments.

### The role of EHD2 in stabilizing membrane curvature at the neck of caveolae

**Fig. 5:**
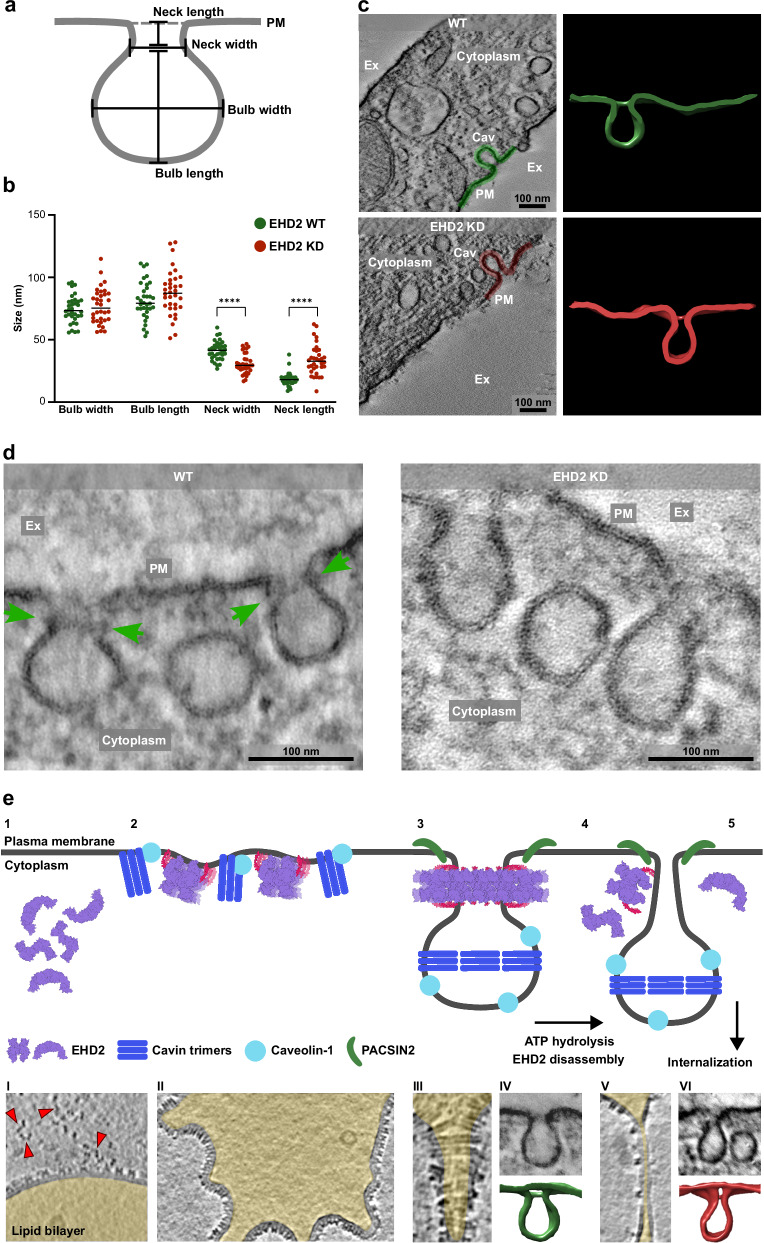
EHD2 serves as a scaffold to direct proper caveolae neck morphology. **a** Schematic representation of caveolae indicating how their morphology was evaluated. **b** Caveolae morphology in the presence (green) and absence (red) of EHD2. Each dot represents an individual caveola, bars represent the mean. Statistical test: two-sided Student’s *t* test or Mann–Whitney rank-sum (see Source Data). *N =* 34 caveolae from 14 tomograms for EHD2 WT and *N =* 34 caveolae from 31 tomograms for EHD2 knockdown (KD) HUVECs for (**b**–**d**) (Supplementary Table 3); *p ≤* 0.0001****. **c** Left—central slices of representative room-temperature tomograms of EHD2 wild-type (WT) and EHD2 knockdown (KD) HUVECs. Right—surface of a segmented caveola is shown; PM: plasma membrane, Ex: extracellular space, Cav: Caveola. **d** Caveolae in EHD2 WT HUVECs (left) exhibit distinct ring-like protein density at the neck (green arrowheads). This density is mostly absent in EHD2 knockdown (KD) HUVECs (left). **e** Model for EHD2 function at caveolae. (top). (1) EHD2 may exist as a cytosolic open dimer. (2) EHD2 is recruited to flat caveolae^47^, adopting the closed oligomeric conformation. The N-terminus acts as a secondary membrane binding site. Cavins and caveolins, possibly assisted by EHD2, initiate caveolar budding^47^. (3) EHD2, together with F-BAR domain protein PACSIN2 and other EHD2-binding partners, shapes the caveolar neck by forming ring-like oligomeric scaffolds. (4) ATP hydrolysis-driven EHD2 disassembly and detachment from the plasma membrane destabilizes the caveolar neck. (5) Loss of the EHD2 scaffold allows internalization of caveolae. Bottom: cryo-ET volume slices of reconstituted EHD2 samples or caveolar morphologies from HUVECs with membrane geometries corresponding to the model. The vesicle’s/tube’s lumen is colored in yellow. I—ATP-bound EHD2 oligomers on a flat membrane surface; non-membrane-bound EHD2 dimers appear in an extended open conformation (red arrowheads, see Supplementary Figs. 1–3); II—flat membrane surface decorated with ATP-bound EHD2 oligomers (see Supplementary Fig. 2); III—EHD2 rings on membrane tubes of varying diameter (see Supplementary Fig. 2); IV—caveola with a regular membrane neck (see **c**); V— thinned membrane tube in a reconstituted EHD2 samples after ATP hydrolysis (see Supplementary Fig. 3); VI—caveola featuring a thin neck in the absence of EHD2 (see **c**).

To relate the structural analysis of the reconstituted EHD2 assemblies to their cellular role at caveolae, we examined caveolar morphology in human umbilical vein endothelial cells (HUVEC) in the presence and absence of EHD2, using small interfering (si)RNA-mediated knockdown. Electron tomograms of semi-thin sections from resin-embedded HUVECs (obtained from a study described in ref. ^29^) were recorded at room temperature, and both the length and the width of the caveolar bulbs and the necks were examined (Fig. 5a and Supplementary Table 3).

In agreement with previous data^29^, knockdown of EHD2 resulted in 2-fold increased caveolae detachment from the plasma membrane (Supplementary Table 3). The absence of EHD2 did not affect the shape of caveolar bulbs. However, the necks of caveolae in EHD2 knockdown cells were significantly narrower and vertically elongated (Fig. 5b, c): In HUVECs, the caveolar necks were 41 ± 8 nm wide and 18 ± 5 nm long, whereas they were 30 ± 8 nm wide and 33 ± 9 nm long in EHD2 knockdown HUVECs (Fig. 5b, c, Supplementary Fig. 8, Supplementary Table 3, and Supplementary Movie 1). These data are consistent with a model in which EHD2 filaments stabilize a defined diameter of the caveolar necks, therefore preventing their detachment from the plasma membrane. Furthermore, many caveolae in EHD2 wild-type (WT) HUVECs exhibited a distinct protein density at the neck region, which were consistent in size and localization with our proposed oligomeric assembly model of EHD2 and in line with previously reported caveolar neck-associated protein densities observed in ultrathin sections of various cell lines^37^. Caveolar neck density was mostly absent or less prominent in EHD2 knockdown HUVECs (Fig. 5d, Supplementary Fig. 8, and Supplementary Movie 2).

## Discussion

Here, we elucidate the structural basis of EHD2 scaffold assembly and show how EHD2 filaments stabilize highly curved membrane tubes mimicking the caveolar neck. We uncover a role for the N-terminal sequence as a spacer between EHD2 filaments, preventing their aggregation and allowing the formation of evenly spaced ring-like structures. Our structural study sheds light on EHD2’s mechanisms as a stabilizer of the caveolar neck.

Membrane-bound oligomeric filaments, such as those of dynamin^38^, Drp1 (ref. ^39^) or OPA1 (refs. ^40,41^), frequently feature helical symmetry, which enables straightforward particle picking in 2D and refinement with helical symmetry and often results in high-resolution reconstructions. In contrast, membrane-bound EHD2 samples were not helical and more heterogeneous, requiring application of cryo-ET and STA analyses to determine their structures. At an average resolution of ~6.7 Å and 10.1 Å for the EHD2 full-length and EHD2^ΔN^ variant, the predominant α-helices in EHD2 could be well resolved, while amino acid side chains were not visible. For the more flexible and therefore less well-defined regions, such as the EH domain and especially the N-terminal sequence stretch, the domain orientation and the course towards the membrane could be deduced. To account for the low resolution of our EM maps, we used tight geometric restraints in a molecular dynamics simulation-based fitting approach of the STA maps. The resulting quasi-atomic models of the EHD2 filaments on membrane tubules allowed us to deduce the molecular basis of the assembly by identifying the participating secondary structural elements, while we could not distinguish individual side chain contacts in the assembly interfaces.

EHD2 oligomerization is driven by the formation of the interfaces previously described for the EHD4 filaments: the EHD-specific dimerization interface in the G-domain, the oligomerization interface formed between the KPF-loop from the G-domain in one dimer and the helical domain of the neighboring dimer, and the canonical G-interface, which involves the surface across the nucleotide binding pockets of neighboring dimers. In the reported EHD2 crystal structure, an arginine residue located at the C-terminus of the EH domain binds into the active site, therefore blocking the formation of the G-interface and oligomerization in an auto-inhibitory fashion^20,28^. In this structure, the position of the EH domains on top of the G-domain is further stabilized by an interaction of a Gly-Pro-Phe (GPF-) motif in the linker between the helical and EH domains, which binds into the canonical NPF-peptide-binding pocket of the EH domain (Supplementary Fig. 1). In the oligomerized EHD2 filament structure, the C-terminal tail of the EH domain needs to be displaced to relieve auto-inhibition of the G-interface. Consequently, specific interactions of the G and EH domains will be destabilized, which would explain the observed large-scale movement of the EH domain on the EHD2 filament. However, in the oligomerized structure, the NPF binding pocket points towards the inside of the filament and may only be partly accessible for peripheral interactions with the described NPF motif-containing binding partners, such as EHBP1, PACSIN2, or MICAL-L1 (refs. ^42–44^). Thus, during complex formation, the EH domain may further reorient, with the NPF-binding pocket directed towards their binding partners.

EHD2 dimers were found to oligomerize in the closed conformation on the lipid surface, with the tip of the helical domains inserting into the membrane. On membranes of low curvature, short oligomeric filaments are heterogeneously oriented, likely since their curvature does not match the curvature of the underlying membrane. On highly curved lipid tubules, EHD2 filaments adopt regular ring-like shapes, suggesting that high membrane curvature drives the formation of regular EHD2 filaments. In turn, EHD2 appears to generate membrane curvature by inserting Phe322 at the tip of the helical domain into the outer leaflet of the membrane bilayer, as demonstrated by mutagenesis and EPR experiments^20,28^. Furthermore, the EHD2 filament acts as a curved membrane scaffold superimposing its curvature on the underlying membrane. In this way, EHD2 scaffold formation and membrane curvature generation are intertwined. Compared to the previously described EHD4^ΔN^ filaments, the angle between assembling dimers is larger in EHD2 filaments, resulting in a higher curvature of the EHD2 scaffolds. This difference angle does not depend on the N-terminus of EHD2, as the EHD2^ΔN^ scaffold stabilizes a similar curvature compared to EHD2. Instead, the difference seems to reflect an intrinsic oligomerization property of individual EHD homologs, for example, by distinct interactions of the KPF-loop in the oligomer. The different filament curvatures may be adapted to the specific cellular sites of action: while EHD4 acts on larger macropinosomes of low membrane curvature, the EHD2 scaffold at the caveolar neck must stabilize a higher membrane curvature.

In addition to the curvature of the membrane tubule, EHD2 oligomers also induce undulations of the membrane surface along the tubule’s axis. Compared to previously proposed models for EHD1 and EHD2 (refs. ^4,28,45^), the membrane-bound EHD2 filaments rest on the troughs, not on the ridges of the undulations. This specific membrane architecture depends on the N-terminus of EHDs. In the auto-inhibited EHD2 structure, the N-terminus of EHD2 is present in a hydrophobic pocket of the G-domain. In the EHD2 filament, we assigned it to a density reaching along the helical domain towards the membrane. This assignment is supported by our previous EPR experiments, indicating membrane insertion of the N-terminus^20^. Furthermore, the low-resolution density at the periphery of the G-domain was absent when the N-terminal stretch was deleted.

The N-terminal truncation had a drastic effect on the overall arrangement of EHD2 filaments. Instead of the more or less regularly spaced rings, filaments now tightly approached each other, similar to the filaments described for EHD4^ΔN^ (ref. ^32^). Deletion of the EHD2 N-terminus also prevented membrane bulging along the axis of the membrane tubule. A related role of the N-terminus as an architectural element was shown for EHD1, as its deletion led to defects in scaffolding, scission and endocytic recycling^4^.

We propose that upon membrane recruitment, the conserved N-terminal sequence switches from the G-domain into the membrane to act as a physical spacer posing steric constraints between adjacent EHD oligomers required for their proper assembly and function. The average distance between adjacent filaments is about 70 Å, with some variations (see Fig. 4a, right, and Fig. 1b). In particular, for shorter distances, the mostly disordered N-terminal sequences from adjacent filaments are sufficiently long to bump into each other inside or outside the membrane, and therefore to prevent the filaments from clashing and maintain a minimal distance. In addition, insertion of the N-terminus into the membrane appears to induce membrane buckles whose positive membrane curvature is opposite to the membrane curvature generated by the tips of the helical domains (see Fig. 4d). Thus, insertion of the helical tip region into or close to the induced membrane buckle may be energetically unfavorable and collision of adjacent filaments may be indirectly prevented via the induced opposite membrane curvatures.

In the cellular environment, the N-terminal spacer may prevent the aggregation of neighboring EHD2 filaments and instead favor the formation of a single EHD2 ring at the caveolar neck. In line with this idea, the EHD2^ΔN^ mutant shows increased caveolar recruitment compared to wild-type EHD2 when transiently expressed in HeLa cells^20,21^. Notably, N-terminal deletion does not affect EHD2’s role in controlling lipid droplet size^24^, which may indicate that such a variant can still form a stable membrane scaffold.

The caveolar neck has a related geometry to the EHD2-coated membrane undulations observed in our cryo-ET reconstruction, featuring a constriction with positive and negative membrane curvature^19,26,46^. Furthermore, the diameter range of the lipid tubules in our in vitro reconstitution is similar to the diameter of the caveolar neck^22,26,27,47^. In the endothelial HUVEC line, we observed significant alterations in caveolar morphology upon EHD2 knockdown only for the caveolar necks, not for the caveolar bulbs. The few caveolae confined to the plasma membrane in EHD2 knockout cells exhibited narrower and more elongated necks compared to those of wild-type cells. This observation corroborates our idea that the reduced overall caveolar mobility^18,22,24,25^ is related to alterations at the caveolar neck where EHD2 serves as a membrane-stabilizing scaffold. Moreover, non-invaginated caveolae^47^ may contain short oligomeric EHD2 filaments, and their oligomerization may support the formation of mature caveolae.

By integrating our structural results on EHD2 with previous structural and biochemical data, we suggest a refined model of the EHD2 cycle during caveolar function (Fig. 5e). In this model, ATP-bound EHD2 dimers are recruited in the open conformation^21^ to flat caveolae at the plasma membrane and assemble into short oligomeric structures. When caveolae invaginate, the short EHD2 oligomers may transition to the closed conformation and form rings surrounding the caveolar neck. Upon membrane binding, the N-terminus switches from the G-domain into the membrane and acts as a spacer preventing the uncontrolled oligomerization of EHD2 filaments. In this way, a single EHD2 ring stabilizes the caveolar neck at its thinnest position to a defined diameter. Oligomerization-dependent ATP hydrolysis may set an intrinsic timer for destabilizing the G-interface, prompting the disassembly of the EHD2 oligomer^4,21,32^. Accordingly, ATP hydrolysis in our reconstituted membrane tubes leads to disrupted EHD2 membrane coating. In the absence of an EHD2 scaffold, the caveolar neck becomes unstable, promoting the detachment of caveolae from the plasma membrane. In this way, ATP hydrolysis in EHD2 may control cellular trafficking of caveolae and, consequently, caveolar density at the plasma membrane^24^. Following dissociation from the membrane, the EHD2 dimer can then switch back to the open conformation in the cytosol to resume a new reaction cycle.

In summary, we used cryo-ET and STA to characterize the structural basis of EHD2 filament assembly and the role of these filaments in stabilizing highly curved membrane tubes mimicking the caveolar neck. Our study lays the groundwork for future in situ approaches aimed at resolving EHD2 structures at native caveolae, potentially capturing additional aspects of the cellular context, such as the presence of cellular interaction partners enriched at caveolae.

## Methods

### Protein purification

Mouse full-length EHD2 (residues 1–543) and EHD2^ΔN^ (residues 19–543) constructs were expressed in *E. coli* (BL21(DE3)-Rosetta2 strain) from a modified pET28 vector as N-terminal His_6_-tag fusions followed by a PreScission protease cleavage site (according to ref. ^28^). Expression plasmids were transformed in *E. coli* host strain BL21(DE3)-Rosetta2 (Novagen). Cells were grown shaking at 37 °C in TB medium. Protein expression was induced by the addition of 40 μM isopropyl-β-D-thiogalactopyranoside (IPTG) at an optical density of 0.6, followed by overnight incubation with shaking at 18 °C. Cells were harvested by centrifugation (5000 × *g*, 20 min, 4 °C) and pellets were resuspended in resuspension buffer (50 mM HEPES/NaOH pH 7.5, 400 mM NaCl, 25 mM imidazole, 2.5 mM β-mercaptoethanol, 250 µM Pefabloc, 1 µM DNase I). Lysis was carried out using a microfluidizer. After centrifugation (40,000 × *g*, 40 min, 4 °C), cleared lysates corresponding to the soluble protein fraction were applied to a Ni-NTA column. The column was extensively washed using washing buffer I (20 mM HEPES/NaOH pH 7.5, 700 mM NaCl, 30 mM imidazole, 2.5 mM β-mercaptoethanol, 1 mM ATP, 10 mM KCl) and washing buffer II (20 mM HEPES/NaOH pH 7.5, 300 mM NaCl, 25 mM imidazole, 2.5 mM β-mercaptoethanol). The protein was eluted using elution buffer I (20 mM HEPES/NaOH, pH 7.5, 300 mM NaCl, 300 mM imidazole, 2.5 mM β-mercaptoethanol). For His-tag cleavage, 150 μg of PreScission protease were used per 5 mg of EHD2 construct. The protein sample was dialyzed overnight at 4 °C against dialysis buffer (20 mM HEPES/NaOH, pH 7.5, 300 mM NaCl, 2.5 mM β-mercaptoethanol) for imidazole removal and then reapplied to the Ni-NTA column for His-tag removal. The protein was eluted in two steps of increasing imidazole concentration using washing buffer II and elution buffer II (20 mM HEPES/NaOH, pH 7.5, 300 mM NaCl, 50 mM imidazole, 2.5 mM β-mercaptoethanol). Concentrated protein was injected into a Superdex 200 gel filtration column, equilibrated with SEC buffer (20 mM HEPES/NaOH, pH 7.5, 300 mM NaCl, 2.5 mM β-mercaptoethanol, 2.5 mM MgCl_2_). A second run of size exclusion chromatography was performed as a polishing step. Fractions containing EHD2 constructs were pooled, concentrated and flash-frozen in liquid nitrogen.

### Liposome preparation

Folch fraction I bovine brain lipids (Sigma) were dissolved in chloroform at a concentration of 25 mg/ml. To form the liposomes, 50 μl of the lipid solution were mixed with 200 μl of a chloroform/methanol (3:1 v/v) solution and dried under an argon stream and inside a desiccator. The lipids were resuspended in liposome buffer (20 mM HEPES/NaOH, pH 7.5, 300 mM NaCl, 1 mM β-mercaptoethanol) and sonicated in a water bath for 30 s.

### Cryo-electron tomography

For the generation of protein-decorated lipid tubules, 80 μM of the indicated EHD2 construct diluted in tubulation buffer (20 mM HEPES/NaOH pH 7.5, 300 mM NaCl, 0.5 mM MgCl_2_) was incubated with 1.125 mM ATP for 5 min at room temperature. Afterwards, Folch liposomes diluted in liposome buffer were added to yield a final concentration of 2 mg/ml. The sample was further incubated for 10 min at room temperature and, prior to plunge-freezing in liquid ethane, 5 nm colloidal gold was added at a 1:40 ratio (v/v). For apo conditions, the 5 min incubation with ATP was omitted. Glow-discharged carbon-coated copper Quantifoil 2/2 grids were spotted with 4 μl of sample, back-blotted for 3 s and plunge-frozen using a Vitrobot Mark II device. Tilt series were acquired using a TFS Titan Krios G3 electron microscope equipped with a Gatan K3 detector and a Bioquantum energy filter and operated at 300 kV in zero-loss mode. The tilt series were collected using the software SerialEM^48^ and in combination with PACEtomo^49^. The nominal magnification was ×42,000, resulting in a pixel size of 1.069 Å in super-resolution mode. Tilt-series were collected from −60° to 60° with a 3° increment, and at a defocus range of −2 to −7 µm, following a hybrid dose scheme^34^. Hybrid tomograms had a zero-tilt image with a total dose of ~20 e^−^/Å^2^, with the remaining dose equally distributed over the remaining images. The total electron exposure per tilt series was 100 e^−^/Å^2^ for full-length EHD2, and 158 e^−^/Å^2^ for EHD2^ΔN^. Tomograms were processed semi-automatically with tomoBEAR^50^, with the key steps including MotionCorrection^51^, CTF determination^52^, fiducial-based tilt series alignment^53^, followed by manual refinement and reconstruction by weighted back-projection in IMOD etomo^54^.

### Subtomogram averaging

For both constructs, subtomograms were picked along the central axis of the lipid tubules using a filament model in Dynamo catalogue^35^ in eight-times binned tomograms (8.552 Å/pix). A total of 14,491 and 30,449 cropped points were defined for full-length EHD2 and EHD2^ΔN^, respectively. The initial coordinates of the subtomograms were imported into SUSAN (github.com/KudryashevLab/SUSAN) and reconstructed with the angular information defined by the filament model. The initial average, which showed cylindrical density, was used as a starting reference for subtomogram alignment and classification. Two iterations with only translational searches and a fixed low-pass filter, followed by 10 iterations with translational and rotational searches and adaptive low-pass filter, were performed. At this stage, different strategies were implemented for each construct. In the case of full-length EHD2, aligned particles were then imported into RELION-4.0^55^ and classified into 8 classes in bin8 with global angular search and a 7.5° step. Four classes (6932 particles) representing ring-like densities of different radii were selected for further processing. Particles were pooled and averaged, followed by symmetry expansion with C8 symmetry to sample the non-aligned parts of the rings, performing subboxing along the ring surface. Half-set identifications of subboxed particles were kept the same as their respective full-ring particles to ensure no spurious correlations in the FSCs. Particles were then re-centered on the ring surface and subjected to auto-refinement in bin8. After this, some subboxed positions converged on the same particles, leaving 44,095 particles after duplicate removal. Consecutive rounds of auto-refinement followed by duplicate removal were performed on bin2 (with and without imposing local symmetry) and unbinned particles, followed by one round of polishing and CTF refinement without high-order aberrations. Final auto-refinement of polished particles in bin1 with C2 symmetry led to a 9 Å resolution map. A final cycle of TomoFrameAlignment and CTF refinement with a tighter mask resulted in an 8 Å resolution map.

A final subset of 37,169 particles before polishing was then converted into a dynamo-style table^56^ and then projected on the high-dose non-tilted images and converted to SPA-style particles STAR file. This was done with the custom script adopted from the hybridSTA^34^ method. These particles were imported into CryoSPARC^57^ and subjected to local refinement with non-uniform filtering, angular search constrained to 1° and translational search to 10 Å. Then, particles were reoriented to match the C2 symmetry axis, and C2 symmetry was applied in all successive alignment rounds. The consensus map of the row of 14 EHD2 monomers was used to focus on the central six monomers of EHD2, and the particle set was expanded by subboxing four monomers on each side, giving a final set of 75,439 particles after duplicate removal. Several rounds of particle subtraction, mask optimization and local refinement were performed. Lastly, the final particle stack was re-imported back into RELION^58^ for reconstruction, postprocessing and local resolution estimation. The final map had a nominal resolution of 6.7 Å.

In the case of EHD2^ΔN^, particle coordinates were projected on high-dose micrographs at 0° tilt using a modified hybridSTA script. Then, particles were extracted, binned 4 times and subjected to 2D classification in RELION^58^ into 50 classes using the default global in-plane angular search. Six classes (1838 particles) were imported into RELION-4.0^55^, auto-refined in bin8 using a spherical mask and global angular search. Afterwards, particles were classified in 3D into 4 classes with a constrained alignment between −45° and 45°. The class (506 particles), which showed a full circle on a Z-slice, was selected and auto-refined. The resulting map was used to subbox particle rows along the pseudo-helix in ChimeraX (UCSF)^59^ using a custom script to keep particle poses. After subboxing, 17,204 particles were reoriented to match the symmetry axis, auto-refined in bin4, bin2 and unbinned with applied C2 symmetry, local angular searches and a mask covering the 12 central monomers in the row. Final auto-refinement of unbinned polished particles with a mask covering the 8 central monomers yielded a map at 13 Å resolution. These particles were then converted into a Dynamo-style table, the coordinates were projected on high-dose non-tilted images, and converted to a SPA-style particles STAR file. This was done with a custom script adopted from the hybridSTA method. These particles were imported into CryoSPARC and subjected to local refinement with nonuniform filtering, C2 symmetry, angular search constrained to 1° and translational search to 8 Å. The final map at 10.1 Å resolution includes eight central monomers. Local resolution was estimated in CryoSPARC^57^.

### Atom model refinement into maps

The atomic models consistent with the cryo-EM maps were generated using MDfit^60^. MDfit uses the cryo-EM map as an umbrella potential to bias (i.e., deform) an underlying structure-based model (SBM)^61^ in order to maximize the cross-correlation between the experimental density and the simulated electron density. An SBM is a molecular force field that is explicitly, albeit not rigidly, biased toward a certain native structure. The SBM for fitting was the EHD2 homo-dimeric crystal structure (4CID) with the sequence homology modeled by Swiss-Model^62^ to remove any missing residues. The portion of the SBM for the KPF loop (residue 110–135), which is missing from the EHD2 structure, is based on the EHD4 crystal structure (PDB 5MVF). Building the SBM from the two crystal structures ensured that the resulting model was maximally consistent with the crystal conformation. This entailed no significant changes in structure as the sequences are highly similar. A preprocessing step was then necessary to move the EH domains within the dimer into a cis positioning because PDB 4CID placed the EH domains in trans. This involved only reorientation of the (421–439 loop); no other residue positions were changed. We refer to this dimeric structure as EHD2-init. An SBM using EHD2-init as the input structure was then generated using SMOGv2.3 (ref. ^63^) with a template called SBM_AA, meaning all non-hydrogen atoms were explicitly represented.

The density corresponding to the central two dimers within the cryo-EM map was chosen as the constraint for MDfit, since this region had the best resolution. Relaxation of the SBM under the influence of the cryo-EM map is performed by molecular dynamics (MD) and, thus, requires an initial condition. Two EHD2-init were rigid-body fit into the map using the “Fit in Map” tool of Chimera. This tetramer includes all studied interfaces and is, in principle, sufficient to model; however, the unfilled electron density due to missing filament neighbors would disrupt the fit. In order to initialize the neighbors on either side of two dimers, the translational symmetry of the filament was exploited. Four additional copies of EHD2-init were added, two positioned on either side, placed such that each dimer-dimer interface was identical. Technically, this was performed by (1) measuring the transformation X between the two central dimers in VMD, (2) duplicating the central dimers, and (3) applying X or -X to the duplicates. This six-dimer system served as the initial condition for MD. Alternating every 10^4^ MD steps, (1) the dynamics were subject to only the SBM and electron density umbrella, (2) additionally, a symmetrizing restraint potential. The symmetrizing restraint potential was implemented by root mean square deviation fitting a central monomer to each monomer and employing weak position restraints. This process allowed the structure to explore the cryo-EM density while additionally maintaining the symmetry of the filament. Through this iterative process, the structure converged within 3 × 10^5^ steps. The middle two dimers were taken as the atomic model. Note that even though the filament’s local C2 rotational symmetry was not explicitly enforced by us during MD, the fact that the SBM was based on a C2 symmetric structure ensured that this symmetry was included. A final energy minimization step was performed in Phenix^64^. For fitting the EHD2^ΔN^ map, the same EHD2 crystal structure-based force field was used, but with the cryo EM-based EHD2 tetrameric model as a starting point. For the final PDB submission, the connecting loops between the helical domain and EH domains and the C-terminal residues of the EH domain were removed due to missing STA density. We maintained these loops in the figures to indicate the connectivity. Residues in the N-terminal loop were truncated to their C_β_ position.

### Resin embedding and sectioning

Resin blocks of HUVEC were used from a previous publication^29^. In short, HUVECs were fixed using 2.5% glutaraldehyde (Sigma-Aldrich G5882-10 ml) and 1% tannic acid in 0.1 M phosphate buffer, pH 7.2, at room temperature for 1 h. After fixation, the cells were washed with 0.1 M phosphate buffer, scraped from the cell culture plates and pelleted by centrifugation at 1000 x *g* for 5 min. HUVECs in suspension were embedded in 1.5% low melting agarose in Milli-Q water. The agarose block was cut into smaller cubes and processed for transmission electron microscopy. The agarose-HUVECs cubes were fixed overnight at 4 °C in 2.5% glutaraldehyde in 0.1 M sodium cacodylate buffer, pH 7. After washing, osmification for 2 h was carried out at room temperature using 1% OsO_4_ in 0.1 M sodium cacodylate, pH 7. Excess osmium was washed using MilliQ water, and samples were incubated for 1 h at 4 °C in 2% uranyl acetate in MilliQ water. Dehydration was carried out using an increasing ethanol series 30% for 15 min, 50% for 30 min, 70% for 30 min, 90% for 30 min, and twice in 100% ethanol for 30 min each step. After dehydration, the cubes were incubated for 15 min in propylene oxide. Infiltration with epoxy resin (Polybed812, Polysciences) was carried out at room temperature by incubating the samples for 40 min in 50% and for 40 min in 70% resin in propylene oxide. Infiltration with 100% resin was carried out overnight at room temperature. Polymerization was carried out for 48 h at 60 °C. Resin blocks were sectioned using a Reichert Ultracut S ultramicrotome and an Ultra 35° diamond knife (Diatome). For ultrastructural morphology assessment, 70 nm sections were collected on in-house prepared Formvar/carbon 100 hexagonal mesh copper grids. Prior to collecting the 150 and 170 nm thick sections for electron tomography, fiducial gold beads were adsorbed onto the grid surface.

### Room-temperature tomography

Tilt series were acquired using a FEI Talos L120C electron microscope equipped with a Ceta detector and operated at 120 kV. All tilt series were collected using the software SerialEM^48^. The nominal magnification was ×42,000, resulting in a pixel size of 3.171 Å. Images were collected from 60° to −60° with a 2° increment and bidirectionally, starting at 0°. Tomograms were reconstructed manually using IMOD^54^. For caveolae morphology analysis and visualization, tomograms were binned four times and reconstructed using a SIRT-like filter with eight iterations. Measurements were done in IMOD. Structures of interest were segmented using Microscopy Image Browser^65^ and Amira (ThermoFisher Scientific). Smoothening of segmented surfaces, visualization and videos were created using Chimera (UCSF) and ChimeraX (UCSF)^59^. Figures 4d, 5a, e, were generated with Inkscape, version 1.4.2.

### Statistics and reproducibility

Data collection and analyses were not performed blind to the conditions of the experiment. Caveolae sample sizes for Fig. 5b are indicated in the respective figure legend. The quantification procedure is described in the respective Methods section together with the software used. For each WT and EHD2 knockdown HUVEC sample, one biological replicate corresponding to one resin-embedded specimen was used. The number of sections analyzed from each resin block is indicated in the legend of Fig. 5b.

For statistical analyses and plotting of Fig. 5b, GraphPad Prism v.7.05 was used. Normal distribution was assessed by applying a D’Agostino–Pearson test. To calculate the significant difference between two groups, normally distributed data was analyzed using an unpaired Student’s *t* test (two-tailed *P*-value); otherwise, a Mann–Whitney rank-sum (two-tailed *P*-value) was used. Differences of *p* ≤ 0.05 were considered significant (*p* ≤ 0.05*, *p* ≤ 0.01**, *p* ≤ 0.001***, and *p* ≤ 0.0001****).

### Reporting summary

Further information on research design is available in the Nature Portfolio Reporting Summary linked to this article.

## Supplementary information

Supplementary Information

Transparent Peer Review File

Reporting Summary

Description of Additional Supplementary Files

Supplementary Video 1

Supplementary Video 2

## Source data

Source Data

## Data Availability

The STA-derived cryo-EM densities of the EHD2 and EHD2^ΔN^ oligomers were deposited in the Electron Microscopy Data Bank (EMDB) under accession codes EMD-53909 and EMD-53911, respectively. Coordinates for the oligomeric EHD2 and EHD2^ΔN^ models were submitted to the Protein Data Bank (PDB) under accession codes 9RBU and 9RCI, respectively. The manuscript refers to the following published structures: 4CID (EHD2 dimer in the closed conformation); 5MTV (EHD4^ΔN^ dimer in the open conformation); 7SOX (EHD4^ΔN^ oligomer in the membrane-bound state). Source data are provided with this paper.
