# Supplementary material for: Structures of EHD2 filaments on curved membranes provide a model for caveolar neck stabilization
Source: Nat Commun. 2026 Jul 31;17:7621. doi: 10.1038/s41467-026-76288-8 (PMC13427840; doi:10.1038/s41467-026-76288-8)
Supplement: Supplementary file 1 — Supplementary Information [file 41467_2026_76288_MOESM1_ESM.pdf]

Supplementary Information for

Structures of EHD2 filaments on curved membranes provide a model for caveolar neck stabilization

Elena Vázquez-Sarandeses, Vasilii Mikirtumov, Jeffrey K. Noel,

Mikhail Kudryashev, Oliver Daumke

This PDF file includes:

Supplementary Figures 1 to 8

Supplementary Tables 1 to 3

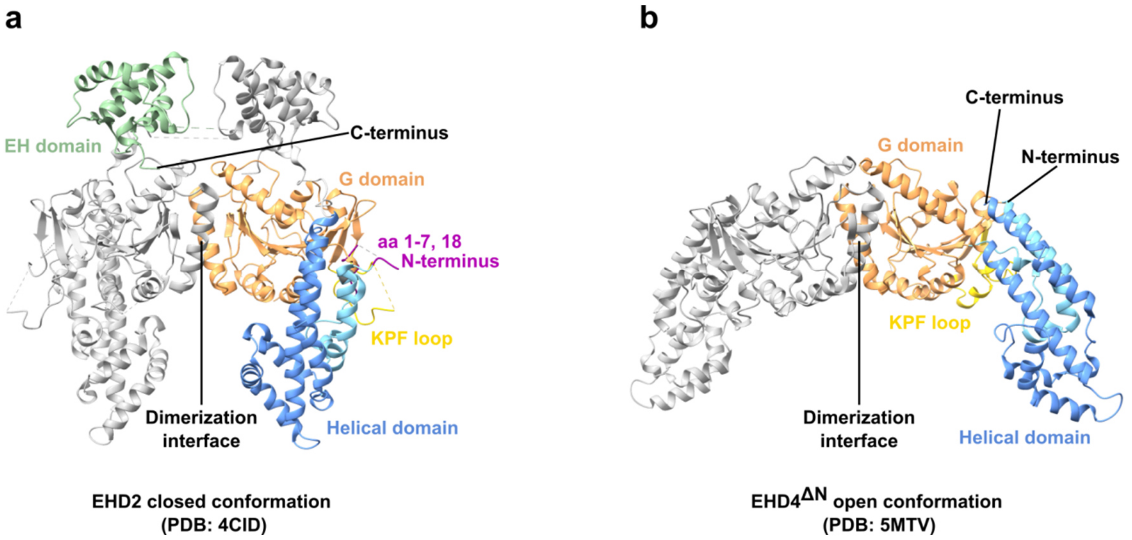

Supplementary Figure 1: Structural overview of the closed and open EHD dimers

a Crystal structure of the EHD2 dimer in the closed conformation (PDB: 4CID). One monomer is colored in gray and the other according to the domain architecture shown in Fig. 1a.

b Crystal structure of the EHD4$^{\Delta N}$ dimer in the open conformation, featuring a 50° rotation of the helical domains compared to the closed state (PDB: 5MTV).

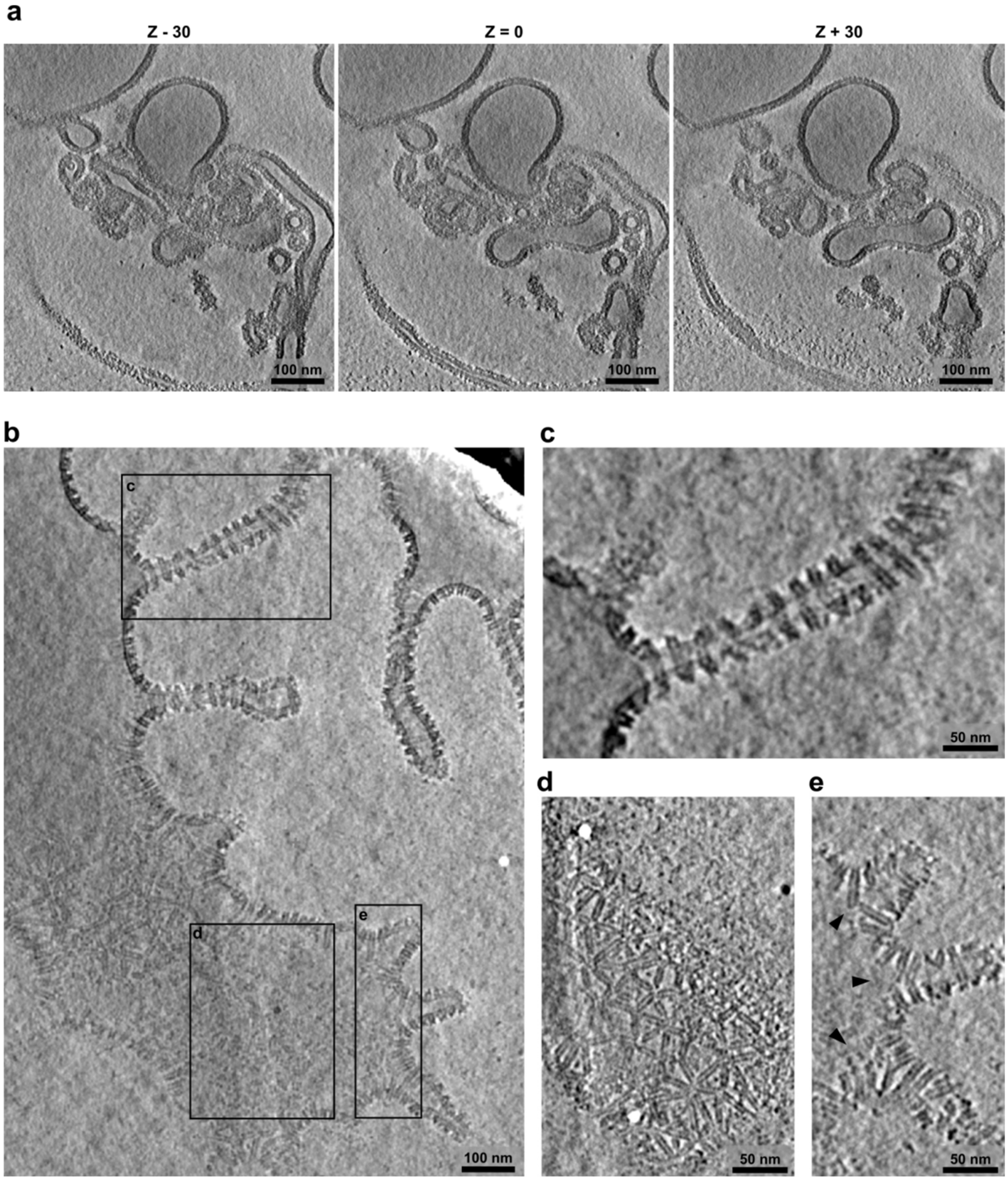

Supplementary Figure 2: EHD2 oligomerizes on lipid bilayers of different curvature in an ATP-binding-dependent manner

a Representative tomogram of nucleotide-free EHD2 reconstituted on liposomes. The middle panel shows the central Z-slice (Z = 0) in which the lumen of the lipid tubule can be discerned. To show that EHD2 cannot organize into regular filaments in the apo state, other Z slices (-30 and +30) showing the surface of the lipid tubules and non-tubulated liposomes are displayed on the left and right panels, respectively.

b Central Z-slice of a representative tomogram showing ATP-bound EHD2 oligomeric filaments of varying lengths on the surface of lipid bilayers of different curvature.

c ATP-bound EHD2 ring-like oligomers on lipid tubules of high curvature.

d ATP-bound EHD2 oligomers on membranes of low curvature.

e Membrane tubulation of EHD2 in presence of ATP occurs in areas of higher curvature where oligomeric filaments encounter each other (arrowheads). Images in panels **c**, **d** and **e** correspond to the inlets highlighted in **b** and show selected Z slices of the tomographic volume.

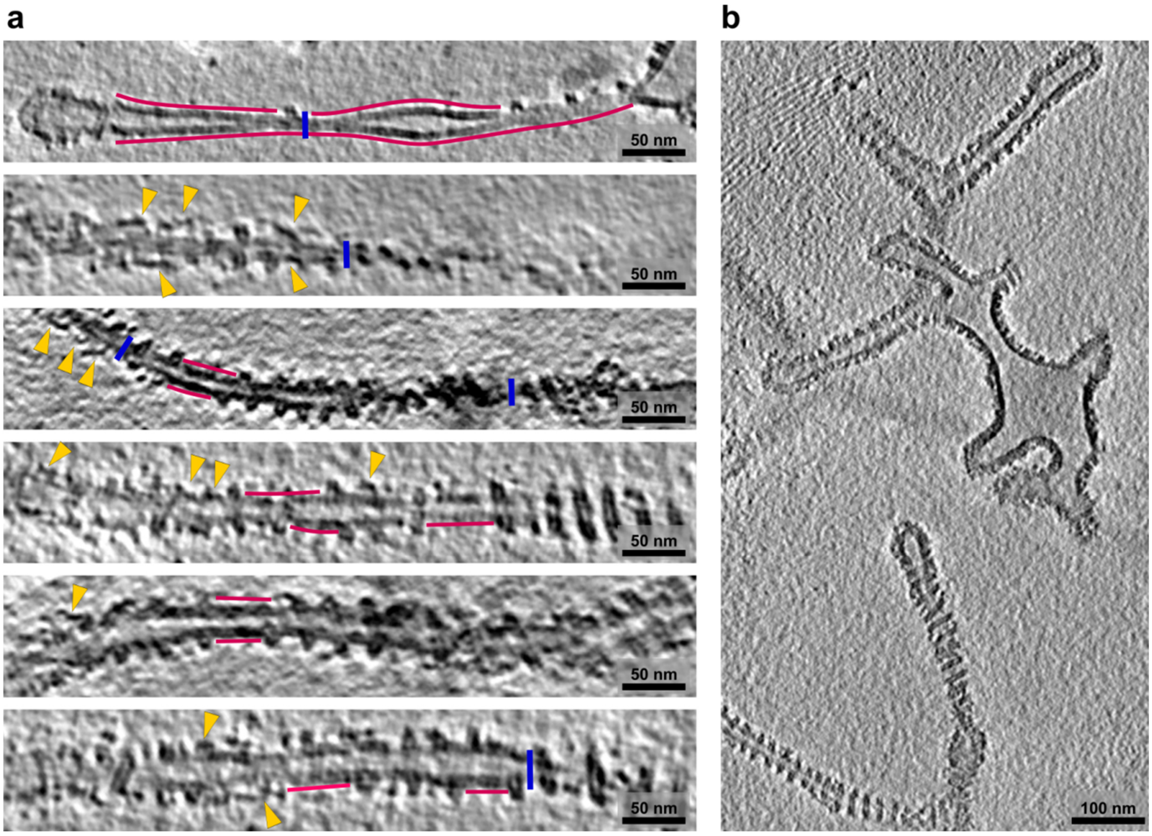

Supplementary Figure 3: ATP hydrolysis leads to oligomer disassembly and membrane destabilization

a Gallery of representative lipid tubules found in tomograms after incubating full-length EHD2 with ATP and liposomes for 120 min. At this time point, about 90% of the ATP is converted to ADP. The central slice of the tomograms is shown. Increased spacing between EHD2 oligomers, interruptions in the protein decoration or almost complete absence of protein were observed (magenta lines). Some areas of the tubules were much thinner or seemed to have collapsed (blue lines). Detached semi-open or open particles were found (yellow arrowheads).
b Lipid tubules with a regular EHD2 decoration were only rarely found under these conditions.

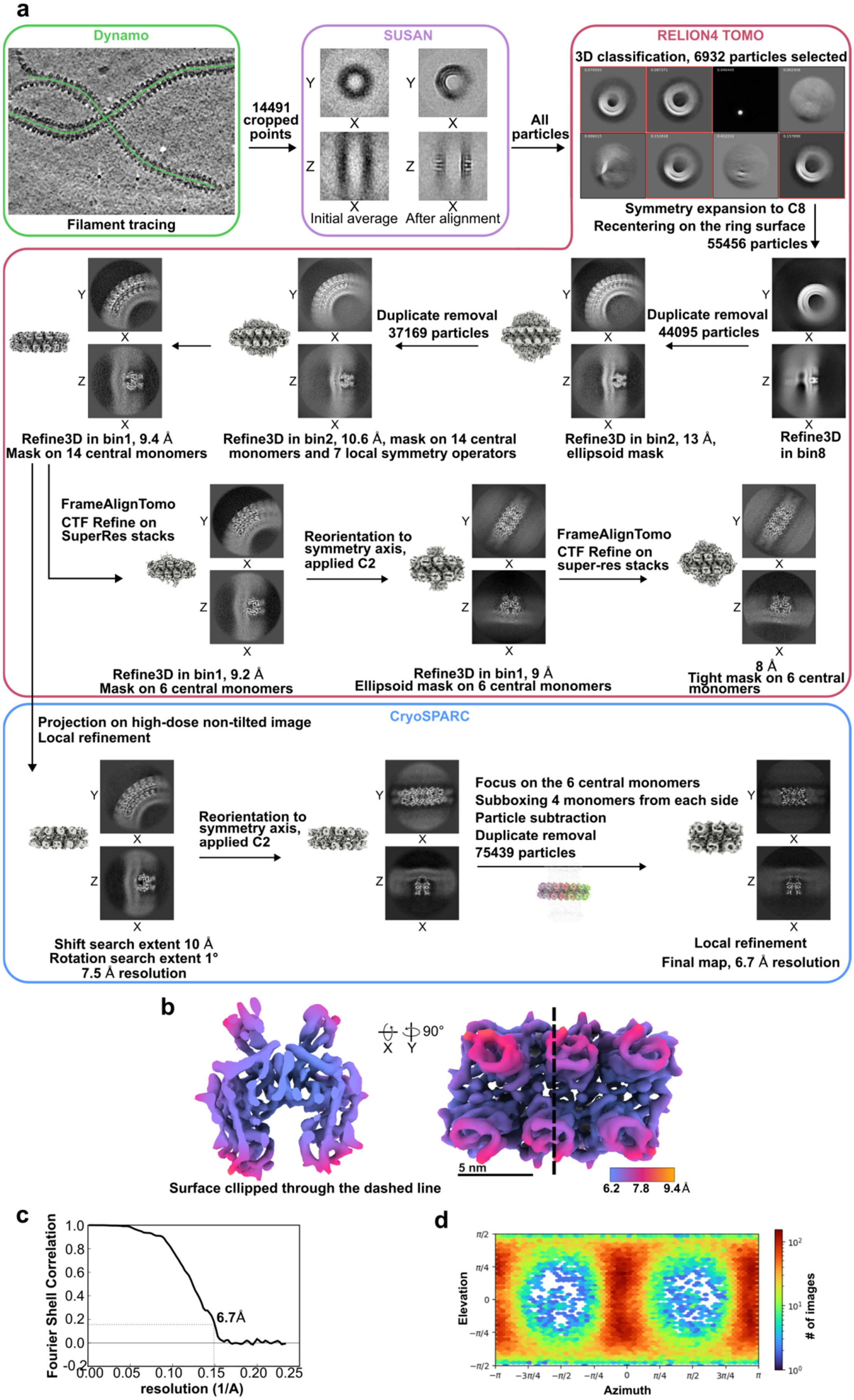

Supplementary Figure 4

Supplementary Figure 4: Subtomogram averaging workflow and structure determination of full-length membrane-bound EHD2

a Cryo-ET and STA data processing flowchart, indicating prominent steps and particle number. Bin1/2/8 - binning by one/two/eight.
b Surface rendering of the final subtomogram averaging map colored according to local resolution. Top and front views are shown.
c The Fourier shell correlation curve, at a cutoff threshold of 0.143, indicates a final overall map resolution of 6.7 Å.
d Angular distribution plot.

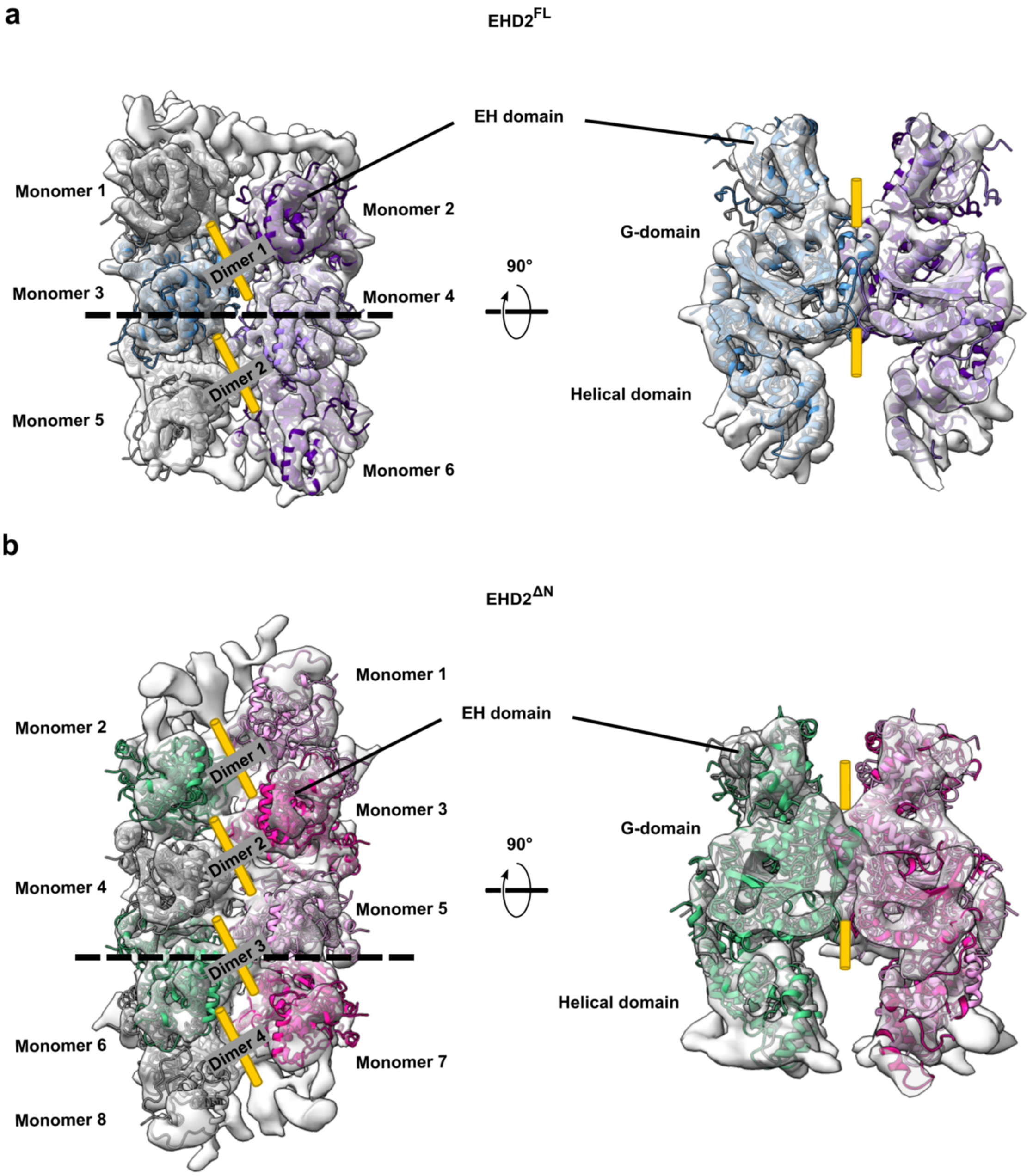

Supplementary Figure 5: Flexible fit of the EHD2 and EHD2$^{\Delta N}$ dimers into the cryo-ET density

The closed EHD2 structure (PDB: 4CID) was fitted into the STA map of membrane-bound full-length **(a)** and N-terminally truncated **(b)** EHD2. The density of the asymmetric unit is shown overlaid with the resulting model from the top (left) and front (clipped, right) views. The dashed lines indicate where the density is clipped. The fit did not require major rearrangements, except for the EH domain and the KPF loop. The yellow tubes indicate the two-fold symmetry axes.

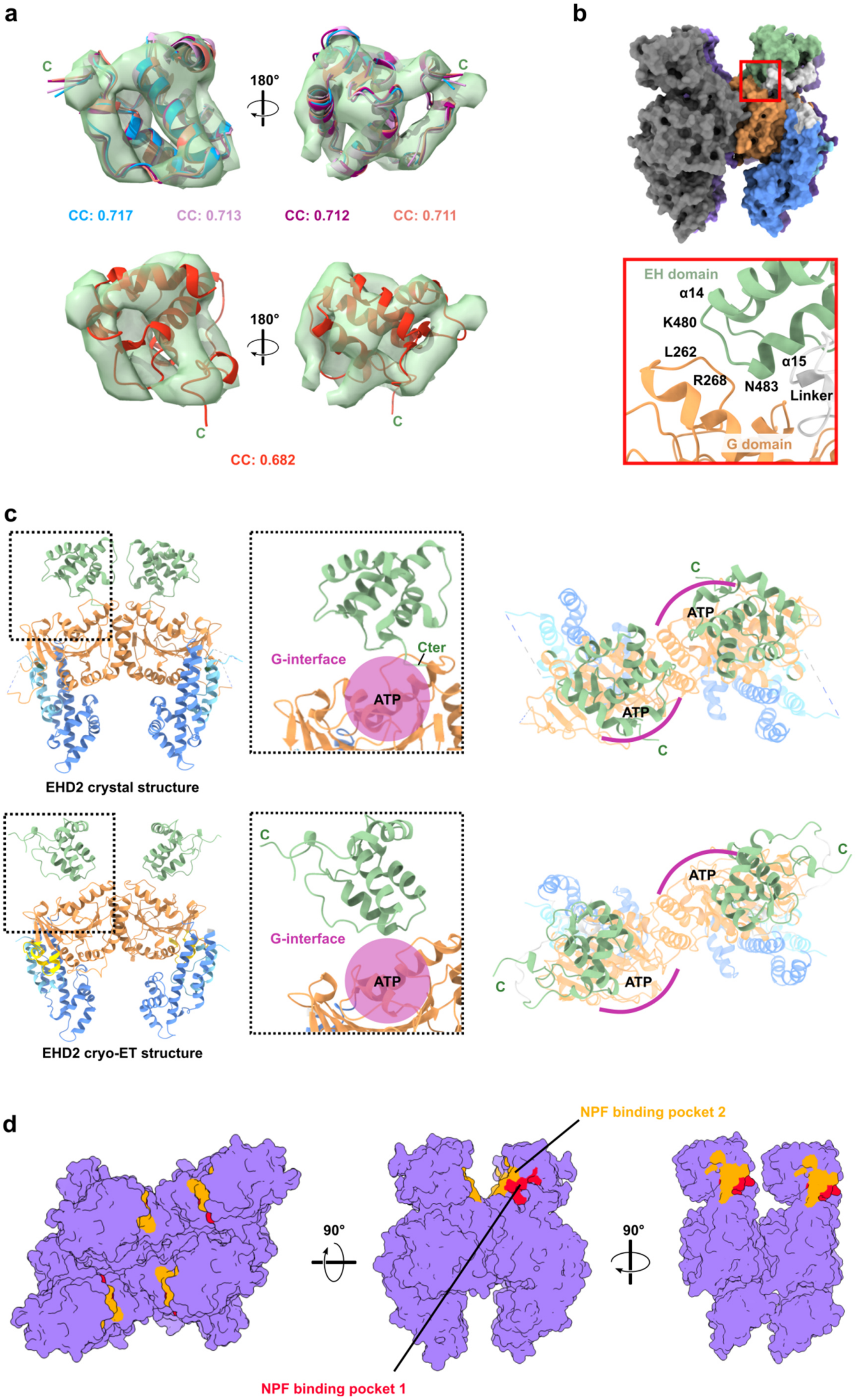

Supplementary Figure 6

Supplementary Figure 6: A new orientation of the EH domain.

a Flexible fitting of seventy different rotations of the EH domain confirms the large-scale domain movement. The best four scoring results, with correlation coefficients (CC) between the map and the model ranging from 0.711 to 0.717, are in the rotated configuration with the C-terminal tail pointing upwards and to the outside of the filament. The crystal structure configuration with the C-terminal tail folding back to the G-domain (red cartoons) does not fit well in the cryo-ET density and resulted in a poorer CC score.
b In the conformation with the highest CC score, the EH domain might generate contacts with the G-domain directly below (red square, magnified).
c Top: The C-terminal tail of the EH domains folds back to the nucleotide pocket of the G-domain in the EHD2 crystal structure (PDB: 4CID), thereby preventing the formation of the G-interface and therefore oligomerization. Bottom: In the new orientation of the EH domain in the cryo-ET structure, the C-terminal tail points towards the outside of the filament and does not prevent the formation of the G-interface. Note that the linker between the helical and EH domain is not shown for visualization purposes.
d The NPF-binding pockets of the EH domains are buried in the filament facing inwards. Accessibility for NPF-motif containing proteins might be compromised in this configuration. In complex with binding partners, the EH domain may therefore adopt yet another conformation.

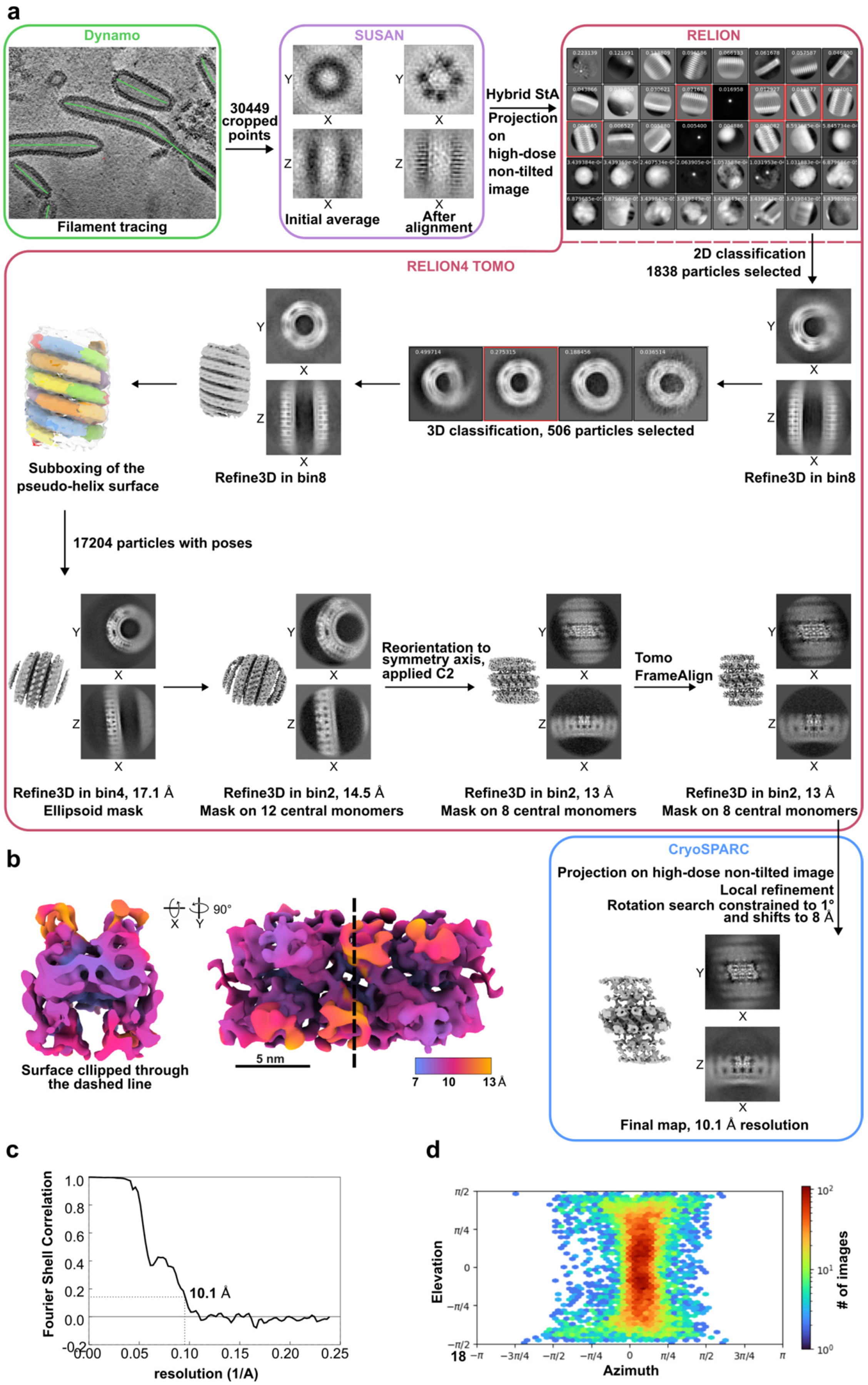

Supplementary Figure 7

Supplementary Figure 7: Subtomogram averaging workflow and structure determination of N-terminally truncated membrane-bound EHD2

a Cryo-ET and STA data processing flowchart, indicating prominent steps and particle number. Bin1/2/8 - binning by one/two/eight.
b Surface rendering of the final subtomogram averaging map colored according to local resolution. Top and front views are shown.
c The Fourier shell correlation curve, at a cutoff threshold of 0.143, indicates a final overall map resolution of 10.1 Å.
d Angular distribution plot.

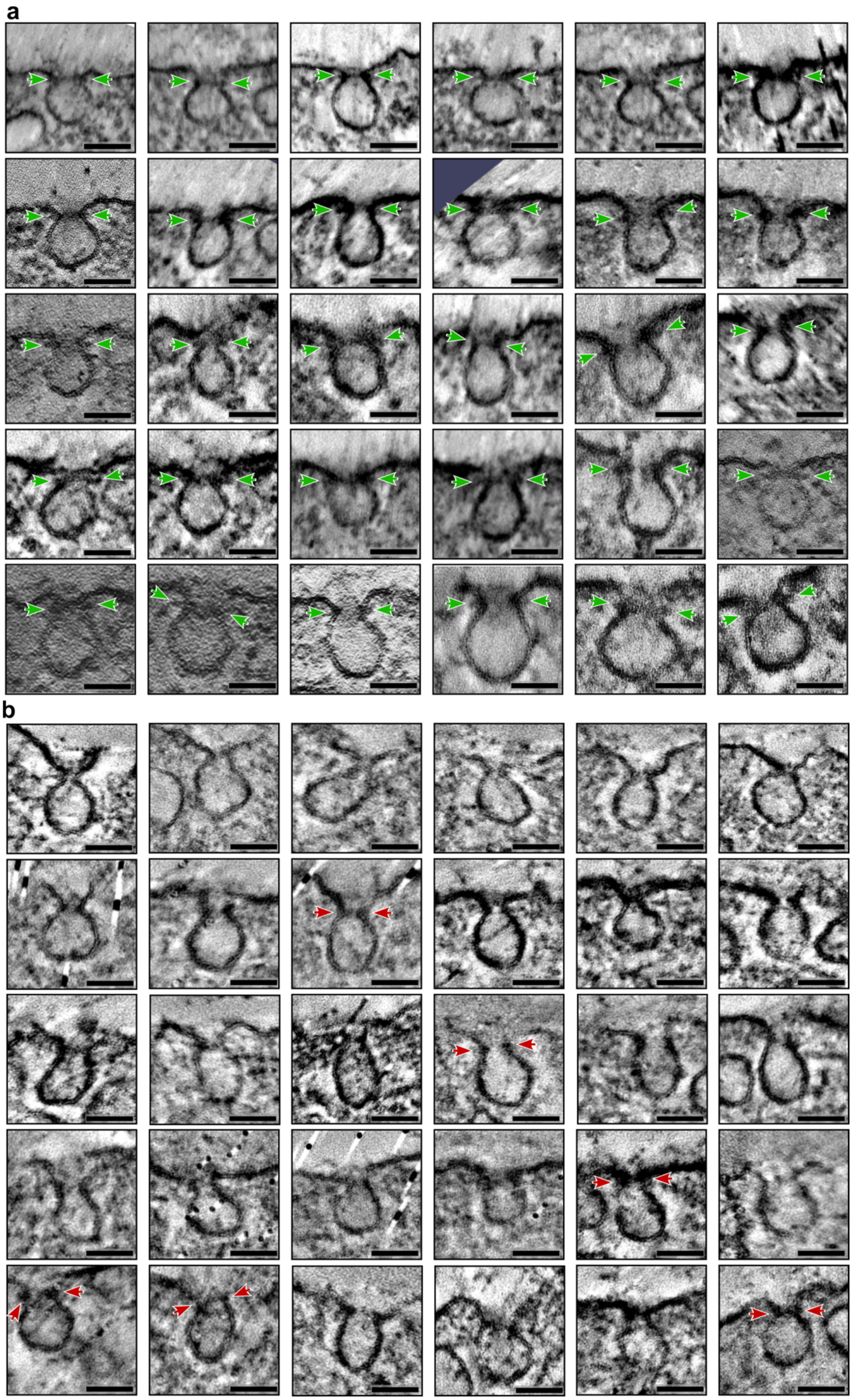

Supplementary Figure 8

Supplementary Figure 8: Gallery of caveolae from EHD2 wild-type and knockdown HUVECs.

a Representative EM micrographs of resin-embedded osmium-stained sections of wild-type HUVECs displaying caveolae. Green arrowheads point to distinct ring-like protein densities routinely observed at the neck of caveolae in these cells.
b In most cases, caveolae from EHD2 knockdown HUVECs did not exhibit a ring-like protein density at their necks. Caveolar necks are vertically elongated, narrower and the membrane appears destabilized. Red arrowheads indicate caveolae displaying some ring-like density. Scale bar: 100 nm.

Supplementary Table 1: Multireference classification and distribution of lipid tubule diameters for full-length and N-terminally truncated EHD2

Full-length EHD2		
Class	Lipid tubule inner diameter (nm)	Number of particles
1	24.4	2,820
2	17.6	1,219
3	23.8	336
4	21.4	648
5	34.3	963
6	27.9	1,525
7	32.6	1,664
8	15.6	2,180
9	24.3	2,741
10	15.8	649

N-terminally truncated EHD2		
Class	Lipid tubule inner diameter (nm)	Number of particles
1	25.5	3,909
2	27.3	8,640
3	28.4	2,739
4	34.9	4,914
5	31.9	5,772
6	29.3	4,071
7	25.3	4,649

N-terminally truncated EHD2 subtomograms were classified using 10 references. Three of the resulting classes, including a total of 17,639 particles, did not yield full cross-sections of lipid tubules and were discarded. Percentages were calculated after removal of these particles.

Supplementary Table 2: Data collection, refinement and validation statistics

	EHD2	EHD2$^{\Delta N}$
**Data collection and processing**		
Magnification	42,000	42,000
Voltage (kV)	300	300
Electron exposure (e^-/Å^2)	100	158
Defocus range (μm)	-2 – -7	-1.5 – -5
Pixel size (Å)	1.069	1.069
Symmetry imposed	C2	C2
Initial particle images (no.)	14,491	30,449
Final particle images (no.)	6,932	17,204
Map resolution (Å)	6.7	10.1
FSC threshold	0.143	0.143
Map resolution range (Å)	6.2 – 9.4	6.3 – 16.6
**Refinement**		
Initial model used (PDB code)	4CID	4CID
Map sharpening *B* factor (Å^2)	-200	-200
Model composition		
Non-hydrogen atoms	16,576	16,576
Protein residues	2084	2084
Ligands	0	0
R.m.s. deviations		
Bond lengths (Å)	0.003	0.003
Bond angles (°)	0.880	0.895
Validation		
MolProbity score[1]	1.31	1.29
Clashcore	2.90	3.57
Poor rotamers (%)	0.28	0.23
Ramachandran plot		
Favored (%)	96.6	97.3
Allowed (%)	3.2	2.4
Disallowed (%)	0.2	0.4

[1]according to:

Williams C.J., et al. MolProbity: More and better reference data for improved all-atom structure validation. *Protein Sci* **27**, 293-315 (2018)

Supplementary Table 3: Analysis of caveolae morphology in the presence and absence of EHD2. Only attached caveolae (att. cav.) connected to the plasma membrane (PM) were considered. The bulb width (B.W.), bulb length (B.L.), neck width (N.W.) and neck length (N.L.) were measured.

EHD2 WT cells							
Tomogram	µm PM	Att. cav. (#)	Cav.	B.W. (nm)	B.L. (nm)	N.W. (nm)	N.L. (nm)
1	1.3	2	1	87	89	47	16
			2	96	91	36	22
2	2.4	1	3	78	81	47	19
3	2.2	3	4	81	99	42	18
			5	69	79	42	16
			6	94	101	44	15
4	2.4	2	7	93	85	50	17
			8	77	111	38	21
5	2.6	3	9	74	83	34	8.9
			10	85	109	60	10
			11	82	96	30	15
6	1.5	1	12	70	75	38	15
7	1.30	1	13	95	110	44	38
8	1.3	2	14	67	75	43	23
			15	72	83	35	18
9	1.3	2	16	57	65	29	12
			17	70	78	27	21
10	1.3	2	18	80	85	42	11
			19	81	96	37	20
11	2.5	4	20	72	70	39	19
			21	78	85	45	20
			22	75	75	54	31
			23	73	78	33	20
12	1.0	9	24	82	79	45	20
			25	69	75	39	16
			26	57	58	48	17
			27	69	76	39	23
			28	56	53	41	20
			29	79	94	34	20
			30	63	68	50	12
			31	56	56	38	18
			32	69	72	31	15
13	0.7	1	33	62	62	43	18
14	1.3	1	34	63	75	55	16
Total	23.2	34					

EHD2 knock-down cells								
Tomogram	µm PM	Attached cav. (#)	Cav.	B.W. (nm)	B.L. (nm)	N.W. (nm)	N.L. (nm)
1	1.3	0					
2	1.4	0					
3	1.2	0					
4	1.3	1	1	60	75	39	29
5	1.3	0					
6	1.3	1	2	89	95	28	61
7	1.3	1	3	90	111	18	52
8	1.5	0					
9	1.3	2	4	65	100	29	20
10	1.0		5	96	128	29	30
11	1.3	2	6	83	89	29	20
			7	72	98	17	44
12	1.4	1	8	115	122	24	42
13	1.1	1	9	67	99	30	8.6
14	1.4	0					
15	1.7	0					
16	1.4	0					
17	1.1	2	10	79	83	27	41
			11	72	93	26	55
18	1.2	4	12	85	93	27	35
			13	78	75	31	35
			14	81	78	22	27
			15	87	103	21	29
19	1.7	3	16	61	88	40	28
			17	65	84	27	28
			18	82	84	42	62
20	1.3	4	19	82	127	21	31
			20	67	69	23	32
			21	94	106	30	33
			22	57	62	28	20
21	1.4	1	23	56	79	44	24
22	1.7	3	24	57	77	30	38
			25	64	87	31	36
			26	72	92	26	42
23	1.7	1	27	87	76	27	34
24	1.8	3	28	66	51	34	35
			29	67	54	35	20
			30	104	95	43	36
25	1.6	1	31	72	70	45	23
26	1.4	1	32	70	90	33	20
27	1.6	0					
28	1.3	1	33	86	86	43	28
29	1.3	0					
30	1.5	0					
31	0.8	1	34	89	67	47	49
Total	42.5	34					
