# Supplementary material for: Structures of EHD2 filaments on curved membranes provide a model for caveolar neck stabilization
Source: Nat Commun. 2026 Jul 31;17:7621. doi: 10.1038/s41467-026-76288-8 (PMC13427840; doi:10.1038/s41467-026-76288-8)
Supplement: Supplementary file 4 — Description of Additional Supplementary Files [file 41467_2026_76288_MOESM4_ESM.pdf]

Description of Additional Supplementary Files

Supplementary Movie 1: A representative tomogram of resin-embedded osmium-stained slices of wild-type HUVECs. Boxed areas displaying caveolae are magnified in the second part of the movie.

Supplementary Movie 2: Representative tomograms of resin-embedded osmium-stained slices of HUVECs, in which EHD2 is knocked down. Boxed areas displaying caveolae are magnified in the second part of the movie.
