# Supplementary material for: Structures of EHD2 filaments on curved membranes provide a model for caveolar neck stabilization
Source: Nat Commun. 2026 Jul 31;17:7621. doi: 10.1038/s41467-026-76288-8 (PMC13427840; doi:10.1038/s41467-026-76288-8)
Supplement: Supplementary file 2 — Transparent Peer Review File [file 41467_2026_76288_MOESM2_ESM.pdf]

nature portfolio

Peer Review File

Structures of EHD2 filaments on curved membranes provide a model for caveolar neck stabilization

Corresponding Author: Professor Oliver Daumke

This file contains all reviewer reports in order by version, followed by all author rebuttals in order by version.

Version 0:

Reviewer comments:

Reviewer #1

(Remarks to the Author)
In this work by Vazquez-Sarandeses, Kudryashev, and Daumke, the structural organization of the EHD2 membrane fission filament is determined using cryo-ET. EHD2 has been shown to be a critical component of caveolae and is thought to be involved in membrane fission, ATP-regulation, stabilization of the organelle at the membrane, and maintenance of the open cavity. The authors present a compelling structure of the filament and identify a key role for a membrane-associated N-terminal domain, which appears to drive the formation of a unique ring-like architecture assembled on membranes. Of interest, the complex associates with regions of negative membrane curvature through the diagonal interaction of two subunits. The authors propose that this stabilizes the neck architecture and could represent the structure found at the base/neck of caveolae in living cells.

Overall, I find this paper to be well done and interesting. It will be valuable to the membrane trafficking field and should help guide future work toward a mechanistic understanding of how EHD2, ATP, and other factors regulate caveolae physiology.

I have a few questions and suggestions that the authors may consider to further strengthen the work. In particular, the paper would benefit from a direct test of the structural model in cells. Specifically, through mutation of the N-terminal spacer and assessment of how this changes EHD2 assembly, as well as caveolae shape and dynamics.

Specific Comments

1. In the structure, there appears to be considerable spacing between each ring—potentially up to ~200 Å. The N-terminal region does not appear to span this entire distance. Could the authors further discuss what determines this regular spacing between rings?

2. There is a large rotation of the EH domain. Can the authors more specifically speculate on how and why this rotation occurs?

3. It is somewhat challenging to orient to the images in Figures 1 and 4. The long-range undulations of the membrane are not immediately clear with the cartoons. I recommend presenting the model in a consistent orientation (e.g., rotated ~90°) to better reflect how the authors envision it assembled on the caveolar neck relative to the plasma membrane. Additionally, showing a repeating unit could help emphasize the long-range undulations across multiple rings. At present, it takes some mental gymnastics to understand how the authors think the assembled units are arranged. More clarity would be helpful for the reader.

4. ATP hydrolysis correlates with increased or disrupted spacing between rings. How might this relate to EHD2 function in cells? Please discuss more in the discussion as it relates to signaling.

5. The work would benefit from a direct mechanistic/functional test of the N-terminal domain and its role in ring spacing and EHD2 assembly at caveolae. For example, expressing an EHD2 mutant lacking this domain in cells (in a knockdown background) could help determine whether it still localizes to caveolae at the plasma membrane and if caveolar shape or dynamics are changed in the absence of this domain.

Reviewer #2

(Remarks to the Author)
Summary:
This manuscript investigates the molecular mechanism by which the ATPase EHD2 stabilizes the neck of caveolae, using the cryo-ET and subtomogram averaging. The identification of the N-terminal sequence as a “spacer” that prevents filament clumping and maintains membrane undulations is an interesting model. While the biological narrative is strong, the reviewer has several technical concerns regarding the structural interpretation.

Strength:
Insight: The identification of the N-terminal sequence as a physical “spacer” provides a novel biophysical explanation for how EHD proteins differentiate in their membrane-remodeling function.

Validation: The use of EHD2-knockout endothelial cells to demonstrate the elongation and narrowing of caveolar necks effectively bridges the gap between in vitro structural observations and in vivo cellular morphology.

Major Concerns:
1. The primary concern lies in the poor fit between the atomic coordinates and the experimental EM density. According to the Map-model fit summary (section 9.5, Page 35) for the N-terminally truncated complex (9RC1):

The overall correlation coefficient is merely 0.42. For a reconstruction reported at 10.1 A, a correlation coefficient of 0.42 indicates that a significant portion of the model may not be accurately supported by the density. The authors are expected to explain how such a low correlation allows for the definition of domain-domain interfaces (e.g., Interface 2).

2. The validation reports show that a large fraction of the model exists outside the experimental density: For 9RC1 (Sec 9.4 Page 34), only 49% of backbone atoms and 44% of all non-hydrogen atoms are inside the map; For 9RBU (Sec 9.4, Page 33), only 54 % of non-hydrogen atoms are inside the map. With more than 50% of the atoms falling outside the map, the “atomic-level” descriptions of protein-protein might not be proper. The authors are expected to clarify which regions lack density and tone down their interpretations accordingly.

3. The PDB validation report itself explicitly states that 'high Q-score values are only expected at resolutions at which atoms can be resolved.' At the reported resolution of 10.1 Å (9RC1), the low Q-scores confirm that individual atoms and residue side-chains are not resolvable. Consequently, the authors' detailed discussion of specific residue-level interactions lacks direct experimental evidence and must be considered speculative. To maintain scientific rigor, the structural analysis should be downgraded to domain-level descriptions and orientations.

Version 1:

Reviewer comments:

Reviewer #1

(Remarks to the Author)
The authors addressed all my previous comments in the revised manuscript. That paper is a valuable contribution to the membrane trafficking and structural cell biology field.

Reviewer #2

(Remarks to the Author)
I thank the authors for their thorough and constructive response. My concerns centered on the risk of over-interpreting a low-resolution STA reconstruction at the atomic/residue level, and the revised manuscript addresses these points appropriately.

The authors now state the local resolution ranges explicitly and acknowledge in the Discussion that individual side-chain contacts cannot be resolved. Removing the side-chain representations from Figs. 2A–C, 3C, 4A and Supplementary Fig. 6B, together with reframing the interface descriptions in terms of secondary-structure elements, resolves my concern. The interface assignments are now presented at a level consistent with the data.

I appreciate that the authors improved the deposited models rather than only the text, removing the unsupported linker loop (415–443), truncating the EH-domain C-terminal residues, and stubbing the N-terminal loop to Cβ ,yielding a measurable improvement in atom inclusion. The rationale for the chosen contour level is reasonable, and providing the maps for threshold inspection is helpful.

I agree with the authors' contextualization of the Q-scores; values near zero are expected at this resolution and are consistent with the secondary-structure-level interpretation now adopted throughout.

The central conclusions, the N-terminal sequence acting as an inter-filament spacer, the larger assembly angle underlying the higher curvature of EHD2 relative to EHD4ΔN, and the resulting caveolar-neck-like membrane geometry, rest on domain-level architecture and the full-length/ΔN comparison, and are well supported by the revised analysis.
I have a minor point to verify before publication:

Supplementary Table 2 lists the EHD2ΔN map resolution as 11 Å, whereas the abstract, main text and Supplementary Fig. 7 report 10.1 Å (FSC 0.143). Please reconcile.

These are trivial to address, and I am supportive of publication.

Open Access This Peer Review File is licensed under a Creative Commons Attribution 4.0 International License, which permits use, sharing, adaptation, distribution and reproduction in any medium or format, as long as you give appropriate credit to the original author(s) and the source, provide a link to the Creative Commons license, and indicate if changes were made.
In cases where reviewers are anonymous, credit should be given to 'Anonymous Referee' and the source.
The images or other third party material in this Peer Review File are included in the article's Creative Commons license, unless indicated otherwise in a credit line to the material. If material is not included in the article's Creative Commons license and your intended use is not permitted by statutory regulation or exceeds the permitted use, you will need to obtain permission directly from the copyright holder.
To view a copy of this license, visit https://creativecommons.org/licenses/by/4.0/

We would like to thank the two reviewers for their positive and constructive feedback. Based on their concerns, we introduced minor changes to our submitted structural models and modified the manuscript at several positions, as indicated in the detailed point-by-point response below. We believe that the revisions have significantly improved the manuscript.

Reviewer #1 (Remarks to the Author):
In this work by Vazquez-Sarandeses, Kudryashev, and Daumke, the structural organization of the EHD2 membrane fission filament is determined using cryo-ET. EHD2 has been shown to be a critical component of caveolae and is thought to be involved in membrane fission, ATP-regulation, stabilization of the organelle at the membrane, and maintenance of the open cavity. The authors present a compelling structure of the filament and identify a key role for a membrane-associated N-terminal domain, which appears to drive the formation of a unique ring-like architecture assembled on membranes. Of interest, the complex associates with regions of negative membrane curvature through the diagonal interaction of two subunits. The authors propose that this stabilizes the neck architecture and could represent the structure found at the base/neck of caveolae in living cells.

Overall, I find this paper to be well done and interesting. It will be valuable to the membrane trafficking field and should help guide future work toward a mechanistic understanding of how EHD2, ATP, and other factors regulate caveolae physiology.
Thank you very much!

I have a few questions and suggestions that the authors may consider to further strengthen the work. In particular, the paper would benefit from a direct test of the structural model in cells. Specifically, through mutation of the N-terminal spacer and assessment of how this changes EHD2 assembly, as well as caveolae shape and dynamics.

Specific Comments
1. In the structure, there appears to be considerable spacing between each ring—potentially up to ~200 Å. The N-terminal region does not appear to span this entire distance. Could the authors further discuss what determines this regular spacing between rings?
Thank you for bringing up this point. The average distance between adjacent filaments is about 70 Å, as we show in Fig. 4A, right; however, it indeed can vary to some extent. In particular for shorter distances, the mostly disordered N-terminal sequences from adjacent filaments are sufficiently long to bump into each other inside or outside the membrane to prevent the filaments from clashing and maintain a minimal distance. In addition, and as we show in Fig. 4, the EHD2 oligomeric rings induce a pattern of positive and negative membrane curvature along the axis of the membrane tube, where N-terminal membrane insertion appears to cause the membrane to buckle along the filament. The positive membrane curvature of the buckle is opposite to the membrane curvature generated or sensed by the tips of the helical domains (which is negative, see Fig. 4D). The insertion of the helical tip region into the buckle may therefore be energetically unfavorable, and collision of adjacent filaments may be indirectly prevented via the induced membrane curvature. We added this idea to the discussion. The corresponding section now reads:

We propose that upon membrane recruitment, the conserved N-terminal sequence switches from the G-domain into the membrane to act as a physical spacer posing steric constraints between adjacent EHD oligomers required for their proper assembly and function. The

average distance between adjacent filaments is about 70 Å, with some variations (see Fig. 4A, right and Fig. 1B). In particular for shorter distances, the mostly disordered N-terminal sequences from adjacent filaments are sufficiently long to bump into each other inside or outside the membrane to prevent the filaments from clashing and to maintain a minimal distance. In addition, insertion of the N-terminus into the membrane appears to induce membrane buckles whose positive membrane curvature is opposite to the membrane curvature generated by the tips of the helical domains (see Fig. 4D). Thus, insertion of the helical tip region into or close to the induced membrane buckle may be energetically unfavorable and collision of adjacent filaments may be indirectly prevented via the induced opposite membrane curvatures.

2. There is a large rotation of the EH domain. Can the authors more specifically speculate on how and why this rotation occurs?
We have previously shown by crystal structure analysis that an arginine residue at C-terminus of the EH domains binds into the active site in the auto-inhibited dimer to prevent EHD oligomerization by blocking the G-interface (see Suppl. Fig. 1). In this way, the position of the EH domain is also stabilized in the auto-inhibited state. When EHD2 oligomerizes on the membrane via the G-interface, the C-terminus needs to be dislodged from the active site to release the G-interface. In the absence of this stabilizing contact, the EH domain may adopt the new position on top of the G domain, as observed in the cryo-EM structure of the EHD2 filament. We expanded the discussion on this point, which now reads:

In the previously determined EHD2 crystal structure, an arginine residue located at the C-terminus of the EH domain binds into the active site, therefore blocking the formation of the G-interface and oligomerization in an auto-inhibitory fashion (Daumke et al., 2007; Shah et al., 2014). The position of the EH domains on top of the G-domain is further stabilized by an interaction of a Gly-Pro-Phe (GPF-) motif in the linker between the helical and EH domains, which binds into the canonical Asn-Pro-Phe (NPF)- peptide-binding pocket of the EH domain (Suppl. Fig. 1). In the oligomerized EHD2 filament structure, the C-terminal tail of the EH domain needs to be displaced to relieve auto-inhibition of the G-interface. Consequently, the specific interaction of the G and EH domain may be destabilized, which could explain the observed large-scale movement of the EH domain on the EHD2 filament. However, in the oligomerized structure, the NPF binding pockets point towards the inside of the filament and may only be partly accessible for peripheral interactions with described NPF-motif containing binding partners, such as EHBP1, PACSIN2, or MICAL-L1 (Giridharan et al, 2013; Guilherme et al, 2004; Senju et al, 2011). Thus, during complex formation, the EH domain may further reorient, with the NPF-binding pocket directed towards their binding partners.

3. It is somewhat challenging to orient to the images in Figures 1 and 4. The long-range undulations of the membrane are not immediately clear with the cartoons. I recommend presenting the model in a consistent orientation (e.g., rotated ~90°) to better reflect how the authors envision it assembled on the caveolar neck relative to the plasma membrane. Additionally, showing a repeating unit could help emphasize the long-range undulations across multiple rings. At present, it takes some mental gymnastics to understand how the authors think the assembled units are arranged. More clarity would be helpful for the reader.
Thanks a lot for the suggestion! Based on this concern, we reordered the representations in Fig. 1D/1E and 4D so that they match the representations in Fig. 4A and adjacent views can

be generated by a single 90° rotation. Furthermore, we now clearly indicate the rotations by arrows. In the graphical representation of Fig. 4D, we added the filament as a repeating unit.

4. ATP hydrolysis correlates with increased or disrupted spacing between rings. How might this relate to EHD2 function in cells? Please discuss more in the discussion as it relates to signaling.
In dynamin superfamily members, GTP/ATP binding stabilizes the switch regions in the active site, therefore facilitating G-domain dimerization via the G-interface. Following ATP/GTP hydrolysis, specific contacts of the switch regions to the γ-phosphate are lost, leading to their destabilization and, consequently, G-domain dissociation. Based on the architecture of the EHD filaments, disruption of the G-interface leads to disassembly of the filaments. The disrupted spacing between EHD2 rings and disrupted coating following ATP hydrolysis is therefore likely related to the disassembly of the oligomers.
In a cellular context, we have previously shown that the absence of EHD2 leads to increased caveolar uptake and higher caveolar mobility. ATP hydrolysis in EHD2 may therefore facilitate caveolar uptake and trafficking in the cell. We updated the corresponding section in the discussion, which now reads:

Oligomerization-dependent ATP hydrolysis may set an intrinsic timer for destabilizing the G-interface, prompting the disassembly of the EHD2 oligomer (Deo et al., 2018; Hoernke et al., 2017; Melo et al., 2022). Accordingly, ATP hydrolysis in our reconstituted membrane tubes leads to disrupted EHD2 membrane coating. In the absence of an EHD2 scaffold, the caveolar neck becomes unstable, promoting the detachment of caveolae from the plasma membrane. ATP hydrolysis in EHD2 may therefore control cellular trafficking of caveolae and, consequently, caveolar density at the plasma membrane (Matthaeus et al., 2020). Following dissociation from the membrane into the cytosol, the EHD2 dimer can then switch back to the open conformation to resume a new reaction cycle.

5. The work would benefit from a direct mechanistic/functional test of the N-terminal domain and its role in ring spacing and EHD2 assembly at caveolae. For example, expressing an EHD2 mutant lacking this domain in cells (in a knockdown background) could help determine whether it still localizes to caveolae at the plasma membrane and if caveolar shape or dynamics are changed in the absence of this domain.
We agree with this idea. We have already previously shown that an EGFP-tagged EHD2$^{\Delta N}$ variant is recruited to a greater extent to caveolae compared to wild-type EHD2 in HeLa cells (see Shah et al, PMID 24508342). These results support our current structural observations, indicating that N-terminal residues control EHD2 oligomerization and that their deletion leads to uncontrolled assembly. In another study, we showed that expression of both EHD2 and EHD2$^{\Delta N}$ can rescue the phenotype of EHD2 knockout mouse embryonic fibroblasts in controlling the size of lipid droplets (Matthäus et al, PMID 32170013), e.g. N-terminal deletion may not affect this functionality of EHD2. We slightly restructured this section in the discussion, which now reads:

In the cellular environment, the N-terminal spacer may prevent the aggregation of neighboring EHD2 filaments and instead favor the formation of a single EHD2 ring at the caveolar neck. In line with this idea, a transiently overexpressed EHD2$^{\Delta N}$ mutant shows increased caveolar recruitment compared to wild-type EHD2 (Hoernke et al., 2017; Shah et al., 2014). Notably,

N-terminal deletion does not affect EHD2's role in controlling lipid droplet size (Matthaeus et al., 2020), which may indicate that this variant can still form a stable membrane scaffold.

As the referee proposed, we also attempted to analyze the consequences of re-expressing the EGFP-tagged EHD2 variant and its N-terminal deletion variant in EHD2 knockdown HUVECs to analyze the consequences for the caveolar neck structure. However, for these experiments, the expression levels of EHD2 need to be vigorously controlled and quantified on an individual cell level to avoid overexpression artefacts prior to EM analysis, and we did not succeed with these quantitative CLEM experiments. We also considered generating a genetically modified HUVEC line with a biallelic truncation of the EHD2 N-terminus. However, applying CRISPR-CAS9 gene editing to a primary human cell line, including the selection of biallelic clones with an in-frame deletion of the EHD2 N-terminus, is a considerable challenge, in particular for our structural biology group, and would require a huge time investment, beyond a normal revision.

Reviewer #2 (Remarks to the Author):

Summary:
This manuscript investigates the molecular mechanism by which the ATPase EHD2 stabilizes the neck of caveolae, using the cryo-ET and subtomogram averaging. The identification of the N-terminal sequence as a "spacer" that prevents filament clumping and maintains membrane undulations is an interesting model. While the biological narrative is strong, the reviewer has several technical concerns regarding the structural interpretation.

Strength:
Insight: The identification of the N-terminal sequence as a physical "spacer" provides a novel biophysical explanation for how EHD proteins differentiate in their membrane-remodeling function.

Validation: The use of EHD2-knockout endothelial cells to demonstrate the elongation and narrowing of caveolar necks effectively bridges the gap between in vitro structural observations and in vivo cellular morphology.
Thank you very much!

Major Concerns:
1. The primary concern lies in the poor fit between the atomic coordinates and the experimental EM density. According to the Map-model fit summary (section 9.5, Page 35) for the N-terminally truncated complex (9RC1): The overall correlation coefficient is merely 0.42. For a reconstruction reported at 10.1 A, a correlation coefficient of 0.42 indicates that a significant portion of the model may not be accurately supported by the density. The authors are expected to explain how such a low correlation allows for the definition of domain-domain interfaces (e.g., Interface 2).
Thank you very much for bringing up this important point! As we show in Supplementary Fig. 4 and 7, the average resolution of our maps ranges from 6.2 to 9 Å for the EHD2 wild-type filaments and 7 to 13 Å for the truncated EHD2 variant which is now explicitly mentioned in the results. At these resolutions, secondary structure can be well discerned (see, for example, Fig. 1D, E), but, indeed, no side chain information. We had already discussed this limitation in the discussion and updated the section to:

At an average resolution of ~6.7 Å and 10.1 Å for the EHD2 full-length and EHD2$^{\Delta N}$ variant, respectively, the predominant α-helices in EHD2 can be well resolved, while amino acid side chains are not visible. For the more flexible and therefore less well-defined regions, such as the EH-domain and especially the N-terminal sequence stretch, the domain orientation and the course towards the membrane could be deduced.

In our fitting approach, we made use of the higher-resolution EHD2 crystal structure and employed a flexible relaxation strategy into the cryo-EM map using tight restraints for maintaining the geometry of the crystal structure. This is evident, for example, by the good quality of the Ramachandran plots in our validation reports (only 1-3 outliers per 530 residues). However, we concur with the referee that we cannot (and, in fact, did not) discuss specific amino acid contacts in the structures and interfaces. We now explicitly acknowledge this limitation in our discussion, which now reads:

To account for the low resolution of our EM maps, we used tight geometric restraints in our molecular dynamics simulations-based fitting approach. The resulting quasi-atomic models of the EHD2 filaments on membrane tubules allowed us to deduce the molecular basis of the assembly mechanism by identifying the participating secondary structural elements, while we could not resolve individual side chain contacts in the assembly interfaces.

In addition to the text changes, we modified the figures to prevent the impression that we deduce residue-residue interactions from our maps.

1. In the legend of Fig. 2A, we included the information that the indicated residues were displayed to mark the beginning and end of the KPF loop and EH domain.
2. We replaced the side-chain representation of Arg19 in Fig. 2B to avoid the impression that we can discern side-chain density.
3. In Fig. 2C, we removed side-chain representations and now solely provide the approximate positions of amino acids within their secondary structure element.
4. In a similar way, we removed the side-chain representation of the N-terminus in Fig. 3C. We clarified in the legend that the N-terminus was included in the model to show that it can reach the membrane.
5. In Fig. 4A, we removed amino acid side chains and replaced them with a loop representation.
6. We also removed amino acid side chains in Supplementary Figure 6B.

2. The validation reports show that a large fraction of the model exists outside the experimental density: For 9RC1 (Sec 9.4 Page 34), only 49% of backbone atoms and 44% of all non-hydrogen atoms are inside the map; For 9RBU (Sec 9.4, Page 33), only 54 % of non-hydrogen atoms are inside the map. With more than 50% of the atoms falling outside the map, the "atomic-level" descriptions of protein-protein might not be proper. The authors are expected to clarify which regions lack density and tone down their interpretations accordingly.
Indeed, the atom inclusion plots in the validation reports indicate that at the recommended contour level (a value provided by us), the wild-type and N-terminal truncated EHD2 models have atoms not accommodated in the cryo-EM density. However, for both maps, we selected high contour levels to highlight the models' fit to regions of defined secondary structure (with resolution ranges of 7-9 Å). In these regions, the density accommodates most of the atoms (see Supplementary Figure 5A, B) at this high contour level. At lower contour levels, the majority of the loops are present in the cryo-EM density.
Besides the measures introduced in the previous response, we now provide the subtomogram average maps and models to the referees so that they can vary the density threshold and judge the validity of our fitting approach.

Finally, we had previously included the connecting loop between the helical domain and EH domains in our atomic models to show the possible connectivity (residues 415-443). However, since there is no supporting density for these loops, we now decided to remove them from the submitted pdb file. Similarly, we truncated the final five amino acids from the EH domain from the model. Furthermore, we stubbed all amino acids of the N-terminal loop at the C_β to acknowledge the fact that only weak density is present for this region. This is now explicitly mentioned in the Methods. These measures increased the map-to-model fit at the same high contour levels by 4% for both structures, for both backbone and all non-hydrogen atoms (see new validation reports).

3. The PDB validation report itself explicitly states that 'high Q-score values are only expected at resolutions at which atoms can be resolved.' At the reported resolution of 10.1 Å (9RC1), the low Q-scores confirm that individual atoms and residue side-chains are not resolvable. Consequently, the authors' detailed discussion of specific residue-level interactions lacks direct experimental evidence and must be considered speculative. To maintain scientific rigor, the structural analysis should be downgraded to domain-level descriptions and orientations.

Thank you, we agree. Q-scores were developed as a metric to analyze the fit of the atomic model to the EM density. In the original report (Pintilie et al, 2020, PMID 32042190), they were benchmarked up to a resolution of 6 Å, while at lower resolution, they are expected to be close to 0, which is the case for our model.

As mentioned already in the previous comments, we have not made statements about residue-level interactions in the manuscript. Furthermore, as recommended by the referee, we now refer in the figures to secondary structure elements rather than to residues and added further modifications to the manuscript, as detailed already in the responses to questions 1 and 2.

Reviewer #1 (Remarks to the Author):
The authors addressed all my previous comments in the revised manuscript. That paper is a valuable contribution to the membrane trafficking and structural cell biology field.
Thank you very much!

Reviewer #2 (Remarks to the Author):
I thank the authors for their thorough and constructive response. My concerns centered on the risk of over-interpreting a low-resolution STA reconstruction at the atomic/residue level, and the revised manuscript addresses these points appropriately.
The authors now state the local resolution ranges explicitly and acknowledge in the Discussion that individual side-chain contacts cannot be resolved. Removing the side-chain representations from Figs. 2A–C, 3C, 4A and Supplementary Fig. 6B, together with reframing the interface descriptions in terms of secondary-structure elements, resolves my concern. The interface assignments are now presented at a level consistent with the data.
I appreciate that the authors improved the deposited models rather than only the text, removing the unsupported linker loop (415–443), truncating the EH-domain C-terminal residues, and stubbing the N-terminal loop to Cβ, yielding a measurable improvement in atom inclusion. The rationale for the chosen contour level is reasonable, and providing the maps for threshold inspection is helpful.
I agree with the authors' contextualization of the Q-scores; values near zero are expected at this resolution and are consistent with the secondary-structure-level interpretation now adopted throughout.
The central conclusions, the N-terminal sequence acting as an inter-filament spacer, the larger assembly angle underlying the higher curvature of EHD2 relative to EHD4ΔN, and the resulting caveolar-neck-like membrane geometry, rest on domain-level architecture and the full-length/ΔN comparison, and are well supported by the revised analysis.
Thank you very much!

I have a minor point to verify before publication:
Supplementary Table 2 lists the EHD2$^{\Delta N}$ map resolution as 11 Å, whereas the abstract, main text and Supplementary Fig. 7 report 10.1 Å (FSC 0.143). Please reconcile.
Thanks a lot for pointing out this error. As shown in Supplementary Fig. 7, the correct value is 10.1 Å. Accordingly, we corrected the value in Supplementary Table 2.

These are trivial to address, and I am supportive of publication.
